# Myristoylation of TMEM106B by NMT1/2 regulates TMEM106B trafficking and turnover

**DOI:** 10.1016/j.jbc.2025.110322

**Published:** 2025-05-30

**Authors:** Alexander Lacrampe, Dan Hou, Isis G. Perez, Belvin Gong, Natalia Franco-Hernandez, Angie Yee, Wenzhe Chen, Maxime Ah Young-Chapon, Hening Lin, Fenghua Hu

**Affiliations:** 1 Department of Molecular Biology and Genetics, Weill Institute for Cell and Molecular Biology, Cornell University, Ithaca, New York, USA; 2 Department of Chemistry and Chemical Biology, Cornell University, Ithaca, New York, USA; 3 Alector Inc, South San Francisco, California, USA; 4 Department of Chemistry and Chemical Biology, Department of Molecular Biology and Genetics, Howard Hughes Medical Institute, Cornell University, Ithaca, New York, USA

**Keywords:** TMEM106B, myristoylation, lysosome, NMT1/2, protein processing, neurodegeneration, protein trafficking

## Abstract

TMEM106B, a type II transmembrane protein localized on the lysosomal membrane, has been identified as a central player in neurodegeneration and brain aging during the past decade. TMEM106B variants that increase TMEM106B expression levels are linked to several neurodegenerative diseases, including frontotemporal lobar degeneration (FTLD). Additionally, the C-terminal lumenal fragment of TMEM106B was recently shown to form amyloid fibrils during aging and neurodegeneration. However, the mechanisms regulating TMEM106B levels are not well understood. Here we show that TMEM106B is myristoylated by NMT1/2 enzymes at its glycine 2 **α**-amino group and its lysine 3 **ε**-amino group. Myristoylation decreases TMEM106B levels by promoting its lysosomal degradation. Furthermore, we demonstrate that TMEM106B C-terminal fragments (CTFs) can be detected under physiological conditions, and the levels of CTFs are regulated by myristoylation and lysosomal activities. In addition, we show that non-myristoylated TMEM106B accumulates on the cell surface, indicating that myristoylation affects TMEM106B trafficking within the cell. Taken together, these findings suggest that TMEM106B myristoylation is an important mechanism regulating its function, trafficking, and turnover.

Transmembrane protein 106B (TMEM106B) has been linked to many brain disorders and brain aging. A large genome-wide association study (GWAS) attempting to identify risk factors for frontotemporal lobar degeneration (FTLD) with TDP43 inclusions uncovered TMEM106B, a protein with unknown function at the time (1). Patients with FTLD develop devastating language disorders and disruptions in their personality and behavior, which severely affect their quality of life (2, 3). The *TMEM106B* risk allele modulates the disease phenotypes of patients with FTLD having mutations in the *granulin (GRN)* gene (1). The main single nucleotide polymorphism (SNP) in *TMEM106B* associated with disease risk, rs1990622, is noncoding and located downstream of *TMEM106B*'s 3′ UTR (1, 4, 5, 6, 7, 8, 9). Since then, this SNP and other non-coding *TMEM106B* SNPs in linkage disequilibrium with rs1990622 have been associated with many neurodegenerative diseases, including FTLD with mutations in the *C9ORF72 gene,* amyotrophic lateral sclerosis (ALS), Alzheimer's disease (AD), Parkinson's disease (PD), chronic traumatic encephalopathy (CTE), hippocampal sclerosis of aging (HS-Aging), and limbic-predominant age-related TDP-43 encephalopathy (LATE) (1, 4, 5, 6, 7, 8, 9, 10, 11, 12, 13, 14, 15, 16, 17, 18, 19, 20, 21, 22, 23, 24, 25, 26). This risk allele is associated with increased TMEM106B levels, suggesting that tight regulation of TMEM106B levels is necessary for healthy brain aging (1, 6, 27, 28). More intriguingly, the C-terminal lumenal domain of TMEM106B has recently been shown to form amyloid fibrils during aging and neurodegeneration (29, 30, 31). In addition to these neurodegenerative diseases, a dominant coding mutation in the lumenal domain of TMEM106B, D252N, was shown to cause hypomyelinating leukodystrophy (HLD) (32, 33, 34).

*TMEM106B* encodes a type II transmembrane protein localized in the lysosome (27, 35, 36), with an N-terminal disordered cytoplasmic domain (37). TMEM106B deficiency in mice leads to lysosomal trafficking defects in the axon initial segment, myelination defects and Purkinje cell death during aging (38, 39, 40). In cell lines, overexpression of TMEM106B causes lysosome enlargement, decreased EGFR turnover, and lysosome acidification defects (35, 38). Taken together, these findings suggest that TMEM106B plays a role in regulating lysosome activities, lysosome size, and lysosome movement and trafficking.

Since increased TMEM106B levels are associated with an increased risk of neurodegeneration, it is crucial to understand how TMEM106B levels are regulated. Previous research has revealed that TMEM106B is turned over in a lysosome-dependent manner (27, 35, 36). In addition, TMEM106B undergoes regulated intramembrane proteolysis (RIP) (41). First, its C-terminal lumenal domain is cleaved by a yet-to-be-identified lysosomal protease (41). Then, its N-terminal fragment (NTF) is cleaved by the intramembrane protease, SPPL2A/B, into a smaller intracellular domain (ICD) (41). This processing might be relevant to the recent findings that aggregates composed of the C-terminal fragment of TMEM106B accumulate in elderly patients with brain disorders (29, 30, 31).

Despite these findings, the molecular mechanisms regulating TMEM106B levels remain poorly understood. Understanding these mechanisms could enable the development of therapeutics to modulate TMEM106B levels. Previously, TMEM106B was identified as a myristoylated protein in wide-scale proteomic screens (42, 43). N-myristoylation is a post-/co-translational lipidation modification in which the enzyme N-myristoyltransferase (NMT) catalyzes the addition of myristic acid to an N-terminal glycine (44). Myristoylation has been shown to affect protein stability, protein–protein interactions, and protein–membrane association (42, 45, 46, 47).

For several decades, NMT was thought to only myristoylate N-terminal glycine (44). However, recent studies have shown that NMT can also myristoylate a lysine side chain if it is immediately after N-terminal glycine. For example, NMT doubly myristoylates the Gly2 and Lys3 of ARF6 to promote its plasma membrane localization (45, 46). This double myristoylation requires a Gly2-Lys3 sequence at the N-terminus. We noticed that TMEM106B also has a Gly2-Lys3 sequence. Thus, in this study, we investigated whether TMEM106B is myristoylated at its N-terminal Glycine 2 and Lysine 3 residues, and how myristoylation regulates TMEM106B.

## Results

### TMEM106B is myristoylated at glycine 2 and lysine 3 residues by NMT1/2

**Figure 1:**
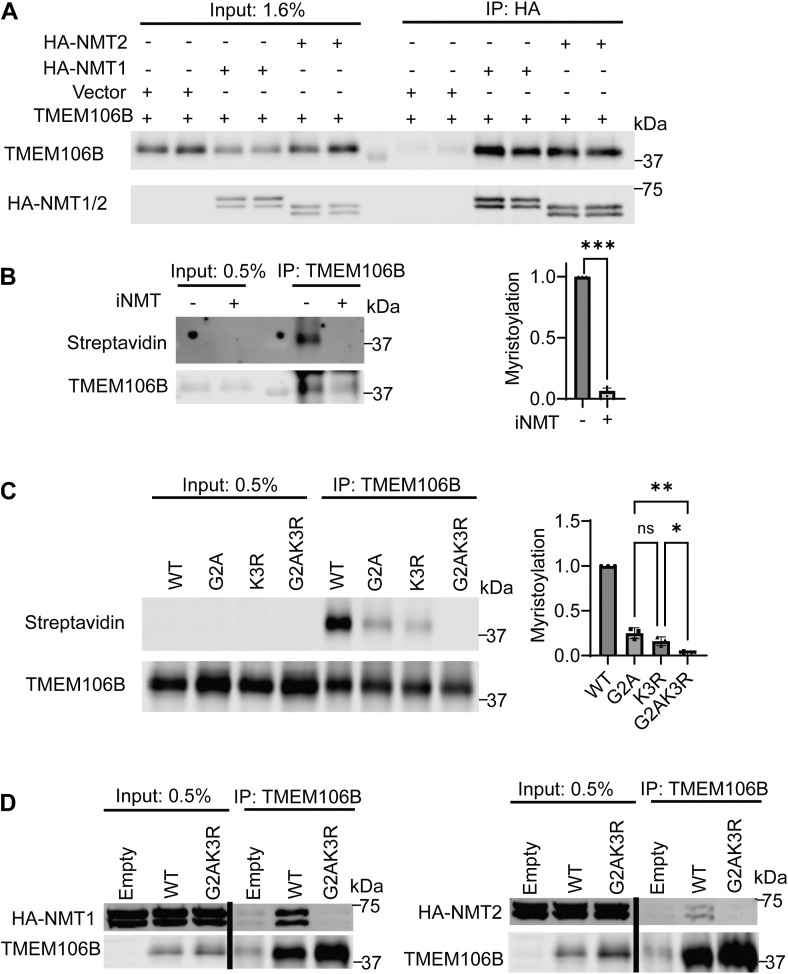
**TMEM106B is myristoylated by NMT enzymes**. *A*, HEK293T cells were transfected with WT TMEM106B and either empty vector, HA-NMT1 or HA-NMT2 as indicated. 48 h after transfection, lysates were immunoprecipitated (IP) using anti-HA antibodies, and the IP samples were analyzed by Western blot. *B*, HEK293T cells were incubated with myristic acid analog with or without NMT inhibitor. TMEM106B was then immunoprecipitated and biotin click chemistry was performed. Proteins were separated using SDS-PAGE and blotted with anti-TMEM106B antibodies or fluorescently labeled streptavidin. The intensity of biotinylated TMEM106B band was quantified and normalized to total TMEM106B levels probed by anti-TMEM106B antibodies (n = 3, one sample *t* test). *C*, HEK293T cells were transfected with WT or TMEM106B mutants as indicated and myristoylation assays were performed as in (*B*) (n = 3, one-way ANOVA with *post hoc* tests). *D*, HEK239T cells were transfected HA-NMT1 And HA-NMT2 along with empty vector, WT, or G2AK3R TMEM106B as indicated. 48 h after transfection the cells were lysed and immunoprecipitated using anti-TMEM106B antibodies. Samples were analyzed *via* western blotting. Data represent mean ± SEM; ns, not-significant; ∗, *p* < 0.05; ∗∗, *p* < 0.01; ∗∗∗, *p* < 0.001; ∗∗∗∗, *p* < 0.0001.

We found that NMT1/2 and TMEM106B physically interact when co-expressed in HEK293T cells (Fig. 1*A*). To confirm TMEM106B myristoylation, HEK293T cells were incubated with a myristic acid analog (Alk12). TMEM106B was immunoprecipitated from the cell lysate, and click chemistry was performed to attach biotin to the myristoylated proteins. Biotinylated proteins were then detected using fluorescently labeled streptavidin. Endogenous TMEM106B in HEK293T cells was found to be myristoylated, which was abolished by treatment with an NMT inhibitor (Fig. 1*B*).

N-terminal glycine and neighboring lysine are the sites for myristoylation in the recently reported ARF6 protein (45, 46). To determine whether TMEM106B is also myristoylated at Gly2 and Lys3, we generated G2A, K3R, and G2AK3R mutants of TMEM106B. These constructs were transfected into HEK293T cells, and myristoylated proteins were labeled with Alk12 and biotin-azide. WT TMEM106B is myristoylated as shown by strong streptavidin signals overlapping with the TMEM106B band detected with anti-TMEM106B antibodies (Fig. 1*C*). The streptavidin signals are significantly reduced by the G2A or K3R mutation and abolished by the G2AK3R double mutation (Fig. 1*C*). G2AK3R mutation also ablates the physical interaction between TMEM106B and NMT1/2 (Fig. 1*D*). These data support that both Gly2 and Lys3 of TMEM106B are myristoylated by NMT1/2.

Sirtuin and HDAC enzymes have been shown to reverse lysine myristoylation by NMT1/2 (45). To test whether Sirtuins can modulate TMEM106B lysine myristoylation, these proteins were co-expressed with TMEM106B in HEK293T cells. Sirtuin overexpression does not affect TMEM106B myristoylation, nor does inhibition of HDAC enzymes with TSA (Fig. S1, *B* and *C*). In addition, TMEM106B and Sirtuins do not co-immunoprecipitate (Fig. S1*A*). These data suggest that TMEM106B is unlikely to be a substrate for Sirtuins or HDACs.

**Figure 2:**
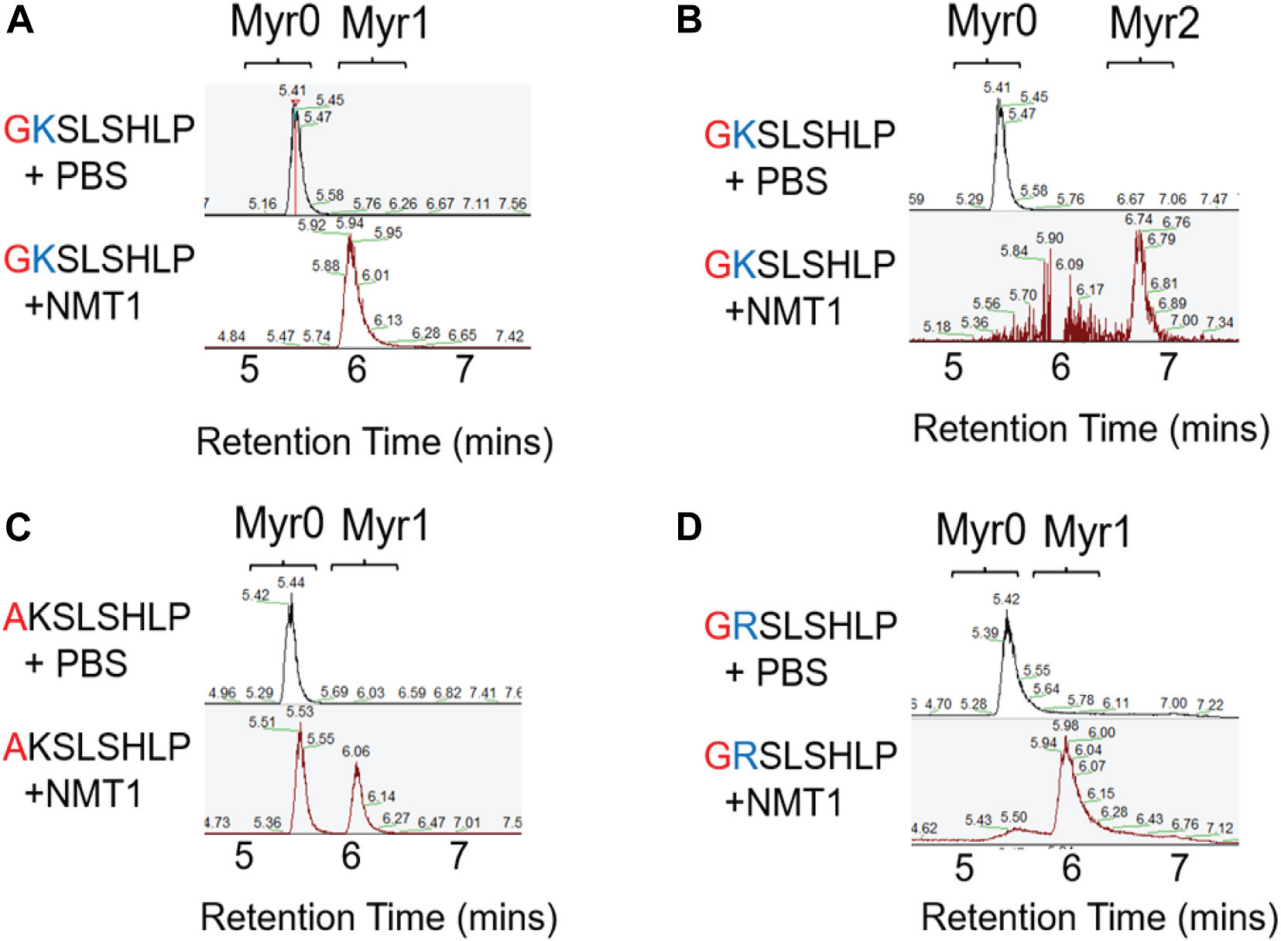
**NMT1 myristoylates TMEM106B peptide *in vitro***. *A–B*, purified NMT1 enzyme was incubated with WT TMEM106BN-terminal peptide and myristoyl-CoA. The reaction products were detected using LC-MS. The ion chromatograms for the substrate and single (*A*) or double (*B*) myristoyl product ions are shown. *C* and *D*, purified NMT1 enzyme was incubated TMEM106B G2A (*C*) or K3R (*D*) peptide and myristoyl-CoA. The reaction products were detected using LC-MS. The ion chromatograms for the substrate and single lysine myristoylated product ions are shown.

We further tested the myristoylation of TMEM106B by NMT1 *in vitro* using purified recombinant enzymes and synthetic peptides derived from the N-terminus of TMEM106B. NMT1 myristoylates WT TMEM106B N-terminal peptide very efficiently with both mono-myristoylation (Fig. 2*A*) and di-myristoylation (Fig. 2*B*) detected. However, di-myristoylated TMEM106B is generated much less efficiently. Using G2A and K3R mutant peptides, we observed strong myristoylation at both K3 and G2 by NMT1 *in vitro* (Fig. 2, *C* and *D*), although to a lesser extent compared to the WT peptide (Fig. 2*A*). This reduction is consistent with our click chemistry experiments (Fig. 1*C*).

### Ablation of TMEM106B myristoylation protects it from lysosome-mediated degradation and increases TMEM106B levels

**Figure 3:**
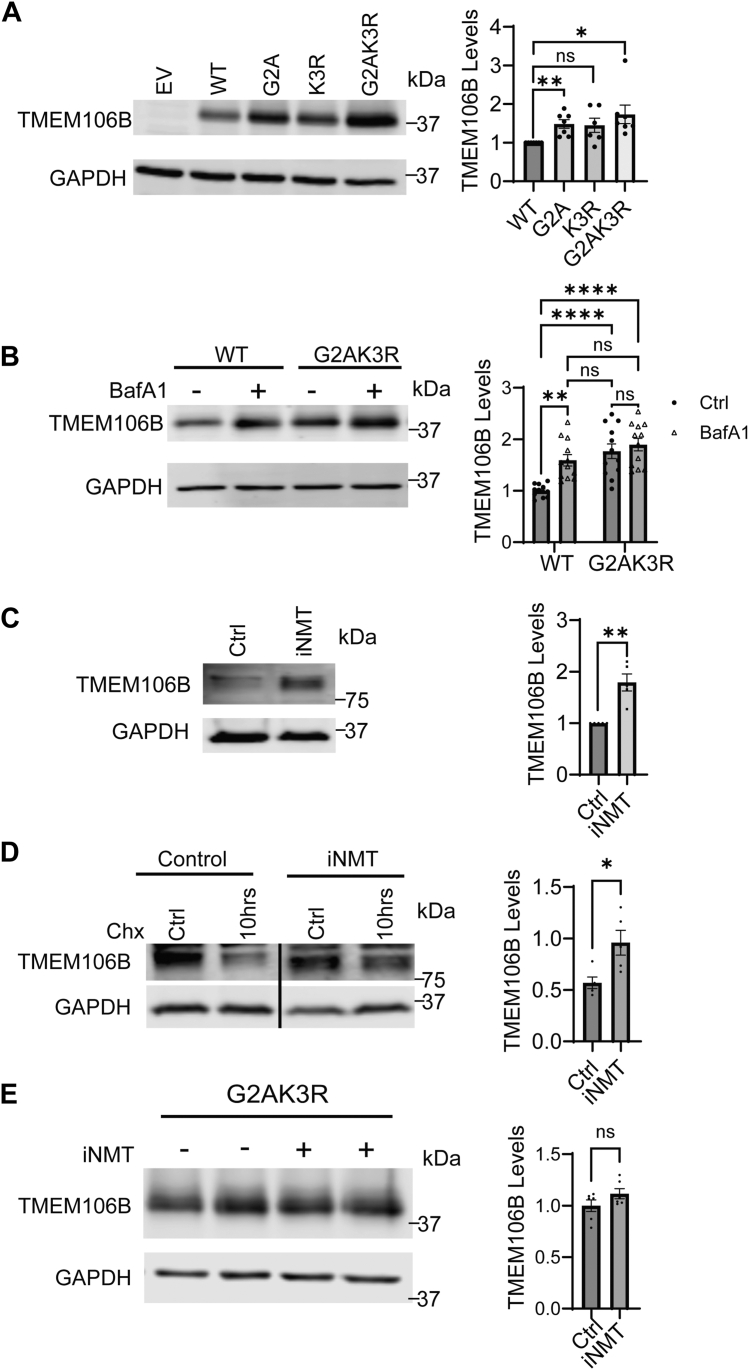
**Ablating TMEM106B myristoylation protects it from lysosomal degradation.***A*, HEK293T cells were transfected with WT, G2A, K3R, or G2AK3R mutant TMEM106B for 48 h. The levels of TMEM106B in the lysates were quantified and normalized to GAPDH (n = 6, one-sample *t* test). *B*, HEK293T cells transfected with WT or G2AK3R TMEM106B for 48 h were either untreated or treated with Bafilomycin A1 (BafA1) for 16 h and then lysed and analyzed with Western blot. The levels of TMEM106B in the lysates were quantified and normalized to GAPDH (n = 12, two-way ANOVA with *post hoc* tests). *C*, HEK293T cells were either treated untreated or treated with iNMT for 24 h before being lysed and analyzed *via* Western blot. The levels of TMEM106B in the lysates were quantified and normalized to GAPDH (n = 5, one-sample *t* test). *D*, HEK293T cells were treated with or without NMT inhibitor (iNMT) overnight and then treated with cycloheximide for 10 h before being lysed and analyzed *via* western blot. The relative levels of TMEM106B to GAPDH were normalized to its levels without cycloheximide treatment in each condition (n = 5, unpaired *t* test). *E*, HEK293T cells transfected with WT or G2AK3R TMEM106B. 24 h post-transfection cells were treated with either iNMT or DMSO control treatment for another 24 h. The levels of TMEM106B in the lysates were quantified and normalized to GAPDH (n = 6, unpaired *t* test) Data represent mean ± SEM; ns, not-significant; ∗, *p* < 0.05; ∗∗, *p* < 0.01; ∗∗∗, *p* < 0.001; ∗∗∗∗, *p* < 0.0001.

While performing TMEM106B click chemistry experiments, we noticed that NMT inhibition or G2AK3R mutation leads to elevated TMEM106B levels. To validate this, we transfected WT, G2A, K3R, and G2AK3R TMEM106B into HEK293T cells. Both G2A and G2AK3R TMEM106B express at levels ∼50% and 90% higher than WT. The K3R mutation shows a trend of increased expression as well but did not reach statistical significance (Fig. 3*A*). Since TMEM106B is subject to lysosome-mediated degradation (35), we hypothesized that Gly2 and Lys3 myristoylation may promote TMEM106B degradation by the lysosome. To test this, we treated cells expressing WT or G2AK3R mutant TMEM106B with bafilomycin A1 (BafA1), an inhibitor of lysosomal acidification and lysosomal function. We found that BafA1 treatment leads to a significant increase in the levels of WT TMEM106B as previously reported but has a minimal effect on the levels of G2A K3R mutant (Fig. 3*B*). Additionally, BafA1 treatment abolishes the difference in the levels of WT and G2AK3R mutant (Fig. 3*B*), supporting that the difference in their levels is caused by lysosome-mediated degradation rather than artifacts from transient transfection.

To further confirm the effect of myristoylation on TMEM106B at endogenous levels, we treated HEK293T cells with an NMT inhibitor for 24 h and found that inhibition of NMT elevates endogenous TMEM106B levels (Fig. 3*C*). Since our antibody has a higher affinity for TMEM106B dimers, which are typically detected under cold and non-reducing conditions (27, 48), we used this condition to detect endogenous TMEM106B. Additionally, we examined TMEM106B stability following the inhibition of protein translation using cycloheximide; inhibition of NMT stabilizes endogenous TMEM106B as shown by a significant decrease in its turnover after cycloheximide treatment (Fig. 3*D*). Finally, treating cells expressing the G2AK3R mutant of TMEM106B with NMT inhibitor does not further increase TMEM106B levels (Fig. 3*E*). Taken together, these findings support that myristoylation promotes degradation of TMEM106B by the lysosome.

**Figure 4:**
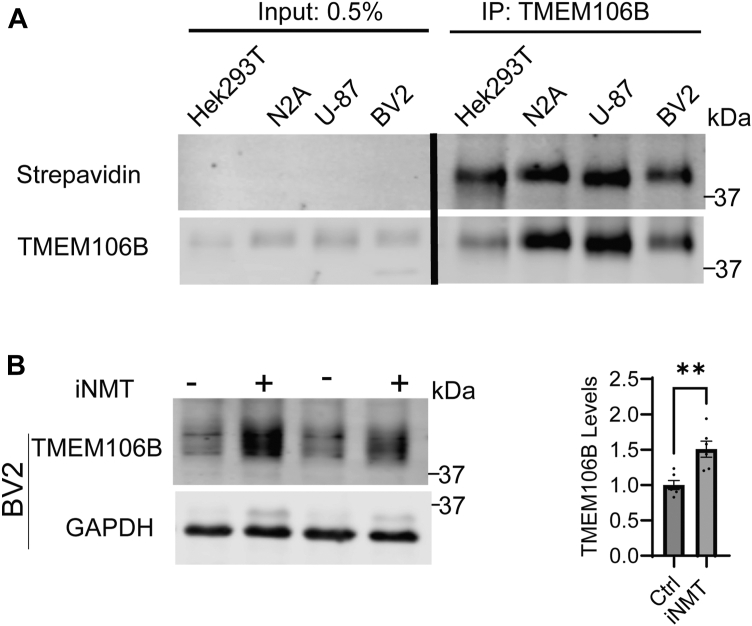
**TMEM106B myristoylation regulates its levels in BV2 cells.***A*, HEK293T, N2A, U-87, and BV2 cells were incubated with myristic acid analog. TMEM106B was then immunoprecipitated and biotin click chemistry was performed. Proteins were separated using SDS-PAGE and blotted with anti-TMEM106B antibodies or fluorescently labeled streptavidin. *B*, BV2 cells were either treated untreated or treated with iNMT for 24 h before being lysed and analyzed *via* Western blot. As previously reported, when kept on ice TMEM106B runs as a monomer in microglia cells (48). The levels of TMEM106B in the lysates were quantified and normalized to GAPDH (n = 6, unpaired *t* test. Data represent mean ± SEM; ns, not-significant; ∗, *p* < 0.05; ∗∗, *p* < 0.01; ∗∗∗, *p* < 0.001; ∗∗∗∗, *p* < 0.0001.

Next, we wanted to confirm that myristoylation plays a role in regulating TMEM106B turnover in cell types relevant to neurodegeneration. We repeated the click chemistry-based myristoylation assay in the neuroblastoma cell line Neuro2a (N2A) as well as the glia cell line U-87 and the microglial cell line BV2. We found that TMEM106B is myristoylated in all three cell lines (Fig. 4*A*). However, inhibition of myristoylation by treating cells with NMT inhibitor causes a significant change in TMEM106B levels in BV2 cells, but not in N2A cells (Figs. 4*B* and S2*A*), although the inhibitor almost abolished myristoylation in N2A cells (Fig. S2*B*).

### TMEM106B myristoylation does not affect its lysosome localization or its ability to induce lysosome enlargement

**Figure 5:**
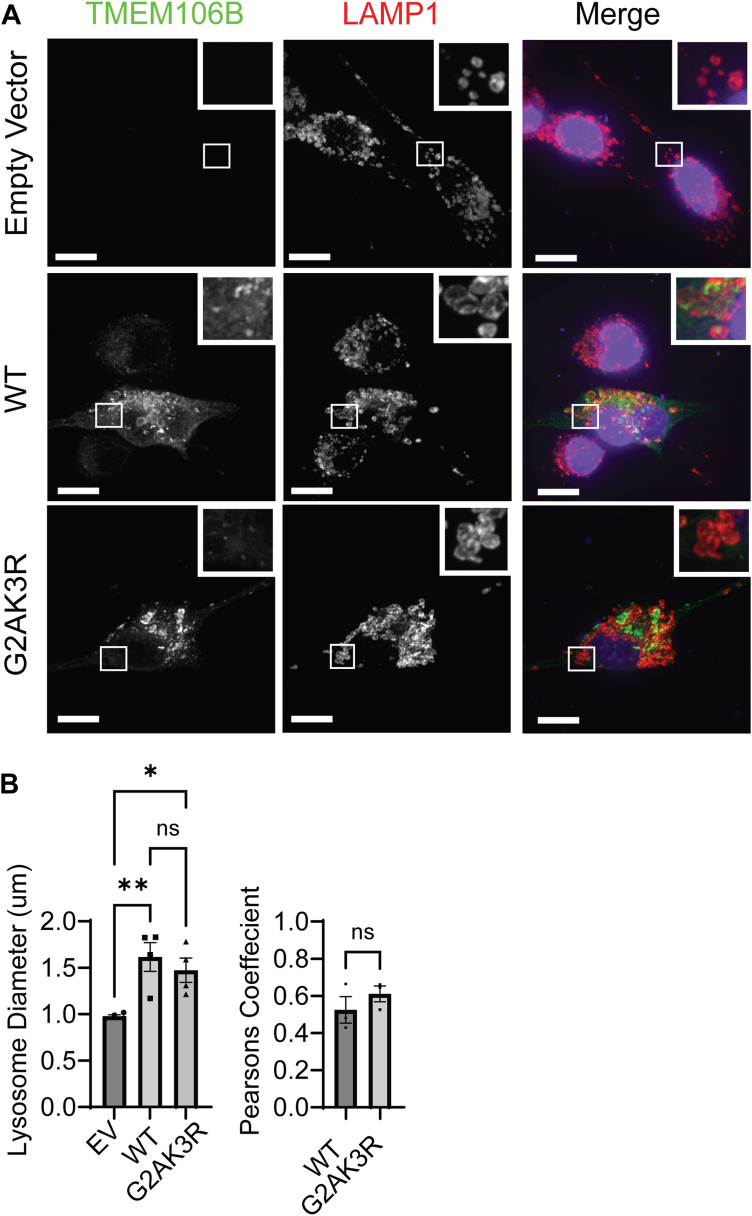
**Myristoylation is dispensable for TMEM106B lysosome trafficking and its ability to induce lysosomal enlargement**. *A*, Neuro-2a cells transfected with empty vector, wild-type TMEM106B, or G2AK3R TMEM106B were fixed and stained with anti-TMEM106B and anti-LAMP1 antibodies. Samples were then imaged using confocal microscopy. Scale bar = 10 μm. *B*, quantification of lysosome enlargement and TMEM106B/LAMP1 co-localization for the experiment in (*A*). For Lysosome diameter, Image J was used to measure the diameter of the five largest lysosomes in each transfected cell. (n = 4, at least 20 cells were quantified per condition per experiment, one-way ANOVA with *post hoc* tests). Image J was used to measure the Pearsons coefficient between the TMEM106B signal and LAMP1 signal (n = 3, at least 20 cells were quantified per condition per experiment, unpaired *t* test =) Data represent mean ± SEM; ns, not-significant; ∗, *p* < 0.05; ∗∗, *p* < 0.01; ∗∗∗, *p* < 0.001; ∗∗∗∗, *p* < 0.0001.

We next investigated whether myristoylation affects the lysosomal localization of TMEM106B. The G2AK3R mutant of TMEM106B co-localizes with the lysosome marker LAMP1, similar to WT TMEM106B (Fig. 5, *A* and *B*), suggesting that myristoylation is dispensable for lysosomal trafficking of TMEM106B. In addition, overnight treatment with NMT inhibitor does not cause a noticeable reduction in lysosomal TMEM106B in HEK293T and HeLa cells (Fig. S3). TMEM106B has been shown to induce lysosomal enlargement when overexpressed (27, 35). We found that overexpression of the G2AK3R mutant in Neuro-2a cells causes a similar level of lysosomal enlargement compared to WT TMEM106B overexpression (Fig. 5*B*), indicating that myristoylation does not affect the ability of TMEM106B to induce lysosomal enlargement.

We also explored whether myristoylation regulates the interactions between TMEM106B and its binding partners. We did not observe any noticeable changes in TMEM106B binding to itself (Fig. S4*A*), or to previously reported binding partners, including lysosome V-ATPase-AP1 subunit and cathepsin D (38, 49, 50) (Fig. S4, *B* and *C*).

### TMEM106B myristoylation regulates the levels of TMEM106B CTFs

**Figure 6:**
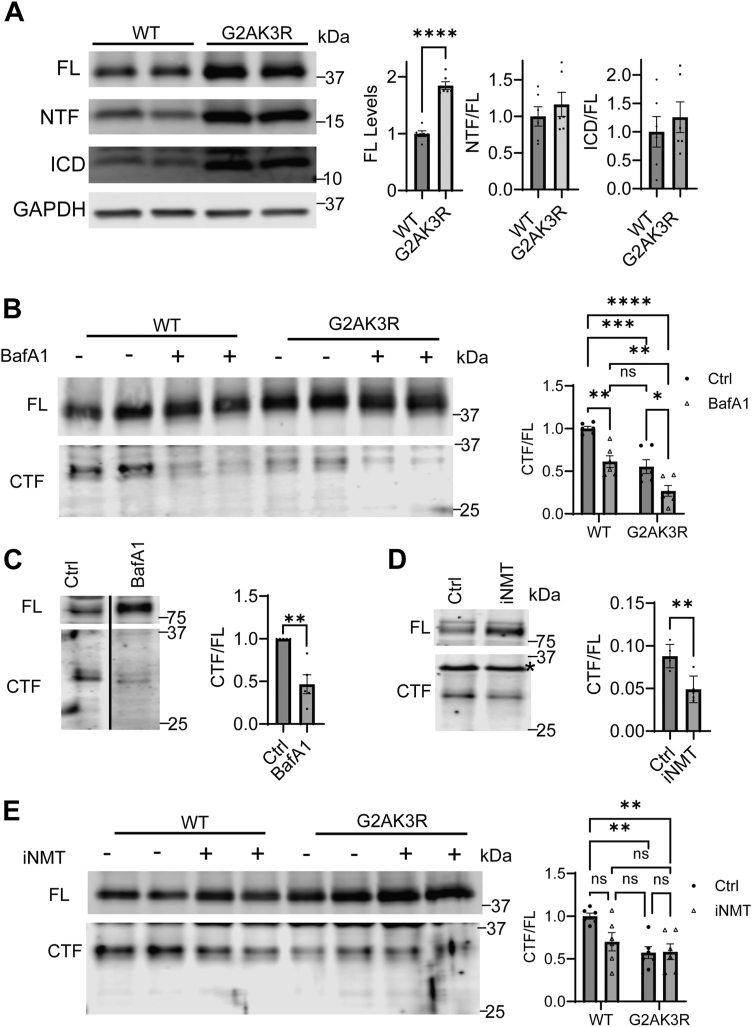
**TMEM106B myristoylation and lysosome pH affect CTF levels.***A*, HEK293T cells were transfected with either WT or G2AK3R mutant TMEM106B. The levels of full-length TMEM06B (FL), N-terminal fragment (NTF), or cytosolic domain (ICD) were analyzed by Western blot using rabbit anti-TMEM106B ICD antibodies 48 h after transfection. TMEM106B levels were quantified by normalizing FL TMEM106B to GAPDH, NTF and ICD levels were normalized to the levels of FL TMEM106B (n = 6, unpaired *t* test). *B*, HEK293T cells were transfected with WT or G2AK3R TMEM106B and were either untreated or treated with bafilomycin A1 (BafA1) for 16 h. The levels of full-length TMEM06B (FL) and C-terminal fragments (CTF) were analyzed by Western blot using human anti-TMEM106B CTF antibodies and quantified (n = 6, two-way ANOVA with *post hoc* tests). *C*, HEK293T cells were treated with BafA1 for 16 h. The levels of full-length TMEM06B (FL) and C-terminal fragment (CTF) were analyzed by Western blot using anti-TMEM106B CTF antibodies and quantified (n = 5, one-sample *t* test). *D*, HEK293T cells were treated with NMT inhibitor (iNMT) for 24 h or untreated. The levels of full-length TMEM06B (FL) and C-terminal fragment (CTF) were analyzed by Western blot using anti-TMEM106B CTF (Clone Ab78) antibodies and quantified (n = 5, unpaired *t* test). *E*, HEK293T cells were transfected with either WT or G2AK3R TMEM106B. 24 h post-transfection cells were treated with either iNMT or DMSO control treatment for another 24 h. The cells were then lysed and analyzed *via* Western blot using CTF antibodies (n = 6, two-way ANOVA with *post hoc* tests). The levels of FL and CTF were quantified and tha ratio between CTF to FL is calculatedData represent mean ± SEM; ns, not-significant; ∗, *p* < 0.05; ∗∗, *p* < 0.01; ∗∗∗, *p* < 0.001; ∗∗∗∗, *p* < 0.0001.

Previously, we showed that TMEM106B undergoes regulated intramembrane proteolysis (RIP) to generate C-terminal fragment (CTF), N-terminal fragment (NTF), and intracellular cytosolic domain (ICD) fragments (41). Recent studies further demonstrated that TMEM106B CTFs form amyloid fibrils during aging and neurodegeneration (29, 30, 31, 51). We, therefore, examined whether myristoylation affects the levels of CTF, NTF, or ICD of TMEM106B. We did not observe any difference in the ratio of NTF or ICD to full-length (FL) TMEM106B in cells overexpressing WT or the myristoylation-defective G2A K3R mutant (Fig. 6*A*). Using a newly developed anti-TMEM106B antibody (Alector clone Ab78) which can specifically recognize TMEM106B CTF at both overexpressed and endogenous levels (Fig. S5, *A* and *C*), we observed a significant reduction in the levels of CTFs in cells expressing the myristoylation mutant G2AK3R compared to WT TMEM106B (Fig. 6*B*). Bafilomycin treatment drastically reduced the levels of CTF generated from overexpressed or endogenous TMEM106B, consistent with our hypothesis that lysosomal proteases are required for TMEM106B cleavage to generate CTF (Fig. 6, *B* and *C*). In addition, we found that NMT inhibition also reduces the levels of endogenous CTFs in HEK293T cells (Fig. 6*D*), consistent with the observation that G2AK3R mutants have lower CTF levels compared to WT. Finally, treating cells expressing the G2AK3R mutant of TMEM106B with NMT inhibitor does not further decrease the CTF/FL TMEM106B ratio (Fig. 6*E*). Taken together, these findings suggest that myristoylation regulates TMEM106B CTF levels.

### TMEM106B myristoylation regulates cell surface TMEM106B levels

**Figure 7:**
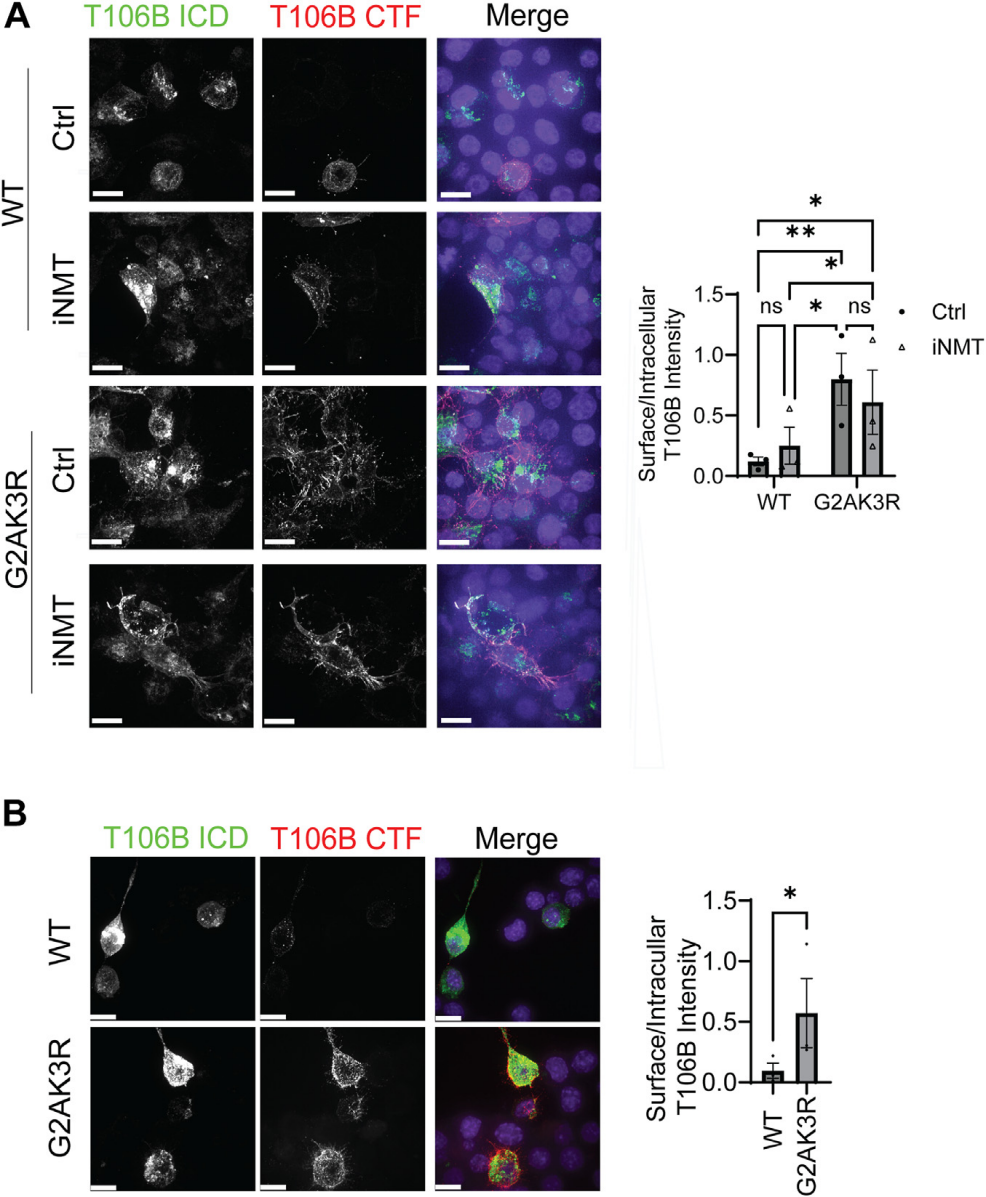
**TMEM106B myristoylationregulates its trafficking to the cell surface.***A*, HEK293T cells were transfected with either WT or G2AK3R mutant TMEM106B. 24 h post-transfection cells were either treated with NMT inhibitor (iNMT) or DMSO control for an additional 24 h before immunostaining. Live staining was performed human anti-TMEM106B CTF antibodies. Cells were then fixed and permeabilzed and immunostained using rabbit anti-TMEM106B ICD antibodies. The ratio of the intensity of cell-surface TMEM106B (T106B CTF) and internal TMEM106B (T106B ICD) was measured per cell using Image J. (n = 3, at least 50 cells were quantified per condition per experiment. Matched two-way ANOVA with *post hoc* tests. Scale bar = 10 μm). *B*, Neuro2A cells were transfected with either WT or G2AK3R mutant TMEM106B and live staining and quantification was performed as in (*A*) (n = 3, at least 30 cells were quantified per condition per experiment. Ratio paired *t* test. Scale bar = 10 μm).

**Figure 8:**
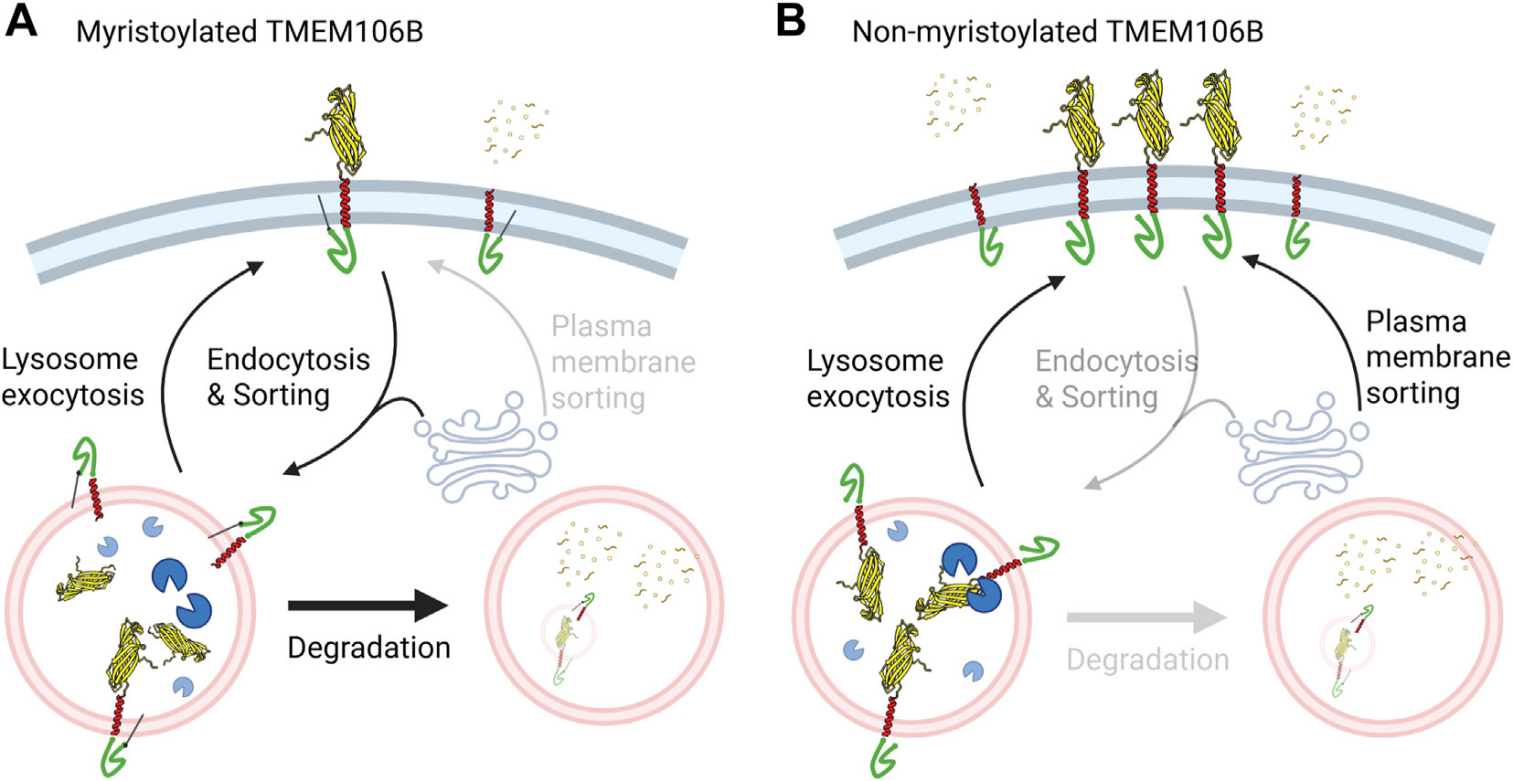
**Model of the role of TMEM106B myristoylation in modulating TMEM106B trafficking and levels**. *A*, when TMEM106B is myristoylated, its levels on the plasma membrane are reduced due to proper sorting to the lysosome at the Golgi or efficient endocytosis from the plasma membrane. Localization on the lysosome membrane renders TMEM106B more likely to be degraded by the lysosome. *B*, when TMEM106B is not myristoylated, it accumulates on the plasma membrane due to miss-sorting to the plasma membrane, increased lysosome exocytosis or defects in its endocytosis. This might lead to decreased CTF production or secretion of CTF into the extracellular space, and less degradation of full-length TMEM106B and NTF by the lysosome. Created with Biorender.com.

Since myristoylation affects TMEM106B turnover and TMEM106B is known to traffic to the cell surface (35), we wondered whether myristoylation regulates TMEM106B trafficking and increases its levels at the cell surface, where it might escape degradation by lysosomal proteases. To test this hypothesis, we transfected HEK293T cells with WT and G2AK3R TMEM106B and performed live cell surface staining. TMEM106B G2AK3R mutant showed a significant ∼five-fold increase in cell surface levels compared to WT (Fig. 7*A*). A trend toward higher cell-surface TMEM106B was observed in cells transfected with WT TMEM106B and treated with NMT inhibitor (Fig. 7*A*). We observed a similar increase in cell-surface TMEM106B when comparing WT and G2AK3R protein in N2A cells (Fig. 7*B*). These data, combined with the observed effect of myristoylation on TMEM106B stability, support a model in which non-myristoylated TMEM106B is protected from lysosome degradation by trafficking to the plasma membrane (Fig. 8).

## Discussion

### Myristoylation of TMEM106B by NMT1/2

Protein myristoylation has been found to occur on both N-terminal glycine and lysine residues in ARF6 (45, 46). In ARF6, the N-terminal glycine is more efficiently myristoylated than the lysine (45). To our knowledge, TMEM106B is the second example of a protein that can be myristoylated at Gly2 and Lys3 (45). Unlike ARF6, both G2 and K3 are similarly important for TMEM106B myristoylation since mutation drastically decreases its myristoylation (Figs. 1 and 2). Furthermore, Sirtuin or HDAC enzymes are unable to remove K3 myristoylation in TMEM106B (Fig. S1). Further studies comparing TMEM106B, ARF6, and other di-myristoylated proteins are needed to determine what features of myristoylated proteins make them substrates for Sirtuin and HDAC enzymes. Interestingly, only the G2A and G2AK3R mutants have significant effects on TMEM106B stability (Fig. 3*A*). This suggests that glycine myristoylation is more important for regulating TMEM106B's stability than lysine's myristoylation. We speculate that an unidentified myristoylation-dependent binding partner of TMEM106B, which regulates its degradation by the lysosome, prefers binding to glycine-myristoylated TMEM106B.

### Myristoylation regulates TMEM106B CTF levels

While our previous work showed that TMEM106B is processed into an N-terminal fragment and then further cleaved by SPPL2A into a cytosolic domain (41), we were unable to detect the formation of a corresponding C-terminal fragment due to the lack of antibodies recognizing CTF domain. Using the new TMEM106B CTF antibody described herein, we have observed the presence of the CTF (Fig. S5, *A*–*C*). We further confirmed that the generation of the CTF depends on lysosomal activities since BafA1 treatment significantly reduced the levels of CTF (Fig. 6, *B* and *C*), consistent with a recent study (52). These CTFs are not detectable in the insoluble fraction (Fig. S5*B*). Taken together, these findings suggest that under normal cell culture conditions, TMEM106B is processed to generate soluble C-terminal fragments, raising the possibility that the insoluble TMEM106B C-terminal fibrils observed in disease states and aging result from non-canonical cleavage of TMEM106B or abnormal aggregation of its CTFs.

Intriguingly, we found that defects in TMEM106B myristoylation dramatically decrease the levels of TMEM106B CTFs (Fig. 6, *B*, *D* and *E*). However, we did not detect any changes in the NTF/FL ratio for the G2AK3R mutant (Fig. 6*A*). The reduction in the CTF but not NTF levels in the G2AK3R mutant could be explained by TMEM106B trafficking defects that deposit full-length TMEM106B and possibly its NTF onto the plasma membrane. This could stabilize the FL protein and NTF and reduce the processing of FL into CTF. Another possibility to explain lower levels of CTFs in the lysate upon myristoylation inhibition is increased secretion of CTFs. However, we failed to detect the presence of CTFs in the cell culture media from cells transfected with WT or G2AK3R mutant (data not shown). The secreted CTFs could be degraded in the extracellular space, and their levels are below the detection limit of our method.

### TMEM106B myristoylation, trafficking, and degradation

Previously, overexpression of TMEM106B has been shown to induce lysosome exocytosis (38, 53). In one study, N-terminal tagged TMEM106B, which cannot be myristoylated, was used (53). Unfortunately, we failed to detect cell surface LAMP1 using cell surface staining and biotinylation in our study (data not shown), thus we cannot determine whether lysosomal exocytosis is affected by TMEM106B. Myristoylation might affect TMEM106B trafficking and turnover through several possible mechanisms. One possibility is that TMEM106B myristoylation could enhance TMEM106B endocytosis to reduce its levels on the plasma membrane. The other possibility is that TMEM106B myristoylation might prevent its mis-sorting to the plasma membrane at the Golgi or through increased lysosomal exocytosis. We have found that the myristoylation defective G2AK3R mutant has increased levels of TMEM106B at the cell surface in both HEK293T and N2A cells (Fig. 7, *A* and *B*). However, inhibition of myristoylation by NMT inhibitors increases endogenous TMEM106B levels in HEK293T, and BV2 cells, but not in N2A cells (Figs. 4, S2). This suggests that N2A cells might have compensatory mechanisms to ensure proper lysosome trafficking of endogenous TMEM106B in the absence of myristoylation modification, but this mechanism is not sufficient when myristoylation-defective TMEM106B is overexpressed. Alternatively, N2A cells might express unique myristoylation-dependent TMEM106B binding partners to stabilize TMEM106B. Loss of these stabilizing interactions could counteract the stabilizing effect of TMEM106B cell-surface trafficking upon treatment with NMT inhibitors. Future work is needed to dissect the mechanisms by which TMEM106B myristoylation regulates its trafficking and levels in different cell types. Additionally, since TMEM106B myristoylation affects its CTF levels, alteration in TMEM106B myristoylation may contribute to the build-up of the TMEM106B CTF fibrils during aging and neurodegeneration. However, it is unclear whether the soluble CTFs observed under normal physiological conditions contribute to fibril formation, or whether TMEM106B fibrils are the result of a different non-canonical processing events.

Very little is known about the role of myristoylation in aging and neurodegeneration. Both NMT1 and NMT2 are expressed most highly during development in mice (54), suggesting that their activity may be reduced in adults. In addition, NMT levels and activity are reduced in T-cells derived from rheumatoid arthritis patients (55), and recent research has linked NMT1 SNPs and reduced NMT1 expression to white matter hyperintensity (WMH) and stroke in the brain (56, 57). Some evidence loosely ties NMT1 to brain aging and neurodegeneration: the rs4341787 SNP located in the enhancer region of the *NMT1* gene modulates AD risk, methylation near the *NMT1* locus is altered during aging, and C9ORF72 dipeptide repeats can bind to NMT enzymes (58, 59, 60). Future studies are needed to determine if NMT activities and TMEM106B myristoylation are altered during aging and neurodegeneration, and if modulating this process has any therapeutic potential.

## Experimental procedures

### Cell culture

HEK293T, BV2, U-87, and Neuro-2a (N2A) cells were maintained in a humidified incubator at 37 °C and 5% CO_2_ and cultured in DMEM media with 10% FBS. Transfections and drug treatment experiments were performed on cells at ∼70% confluency. Transfections were performed as previously described (35, 61). In brief, transfections were performed by mixing 1ug of DNA with 100ul of DMEM and 5ul of polyethylenimine (PEI). The mixture was incubated at room temperature for 15 min before being added dropwise to the cells. 100ul of mixture was used for 3.5 x 10^5^ HEK293T cells and 7 × 10^5^ N2A cells. Cell media was changed 16 h later to remove PEI. To generate CRISPR-Cas9 TMEM106B Ctrl and knockout cells, HEK293T cells were infected with control lentivirus or lentivirus with guide RNA targeted to TMEM106B. Two days later they were selected with 4ug/ml puromycin. When indicated, cells were treated with 20 nM bafilomycin A1 for 16 h, 20 μg/ml cycloheximide for 10 h, 2 μM Trichostatin A (TSA, an HDAC inhibitor), and 2.5 μM DDD86481 (NMT inhibitor) for 2 h for click chemistry experiments and 24 h for other experiments. Cells were tested for *mycoplasma* bi-weekly and are *mycoplasma* free to the best of our knowledge.

### Antibodies and chemicals

The rabbit anti-TMEM106B cytosolic domain antibodies were previously described (35). Chimeric Human/Mouse anti-TMEM106B antibody generation was previously described (62). Clone Ab78 was selected for its ability to detect the full-length and C-terminal fragment (CTF) forms of TMEM106B by immunoblot. Cell lysates from TMEM106B-overexpressing A549 cells and recombinant TMEM106B CTF were used. Specificity was validated by the absence of signal in TMEM106B-KO A549 cells (data not shown). In addition, the following commercial antibodies and chemicals were used: Mouse anti-GAPDH: Proteintech 60004-1-Ig, Mouse anti-HA: Sigma H9658, Mouse anti-hLAMP1: Bd 555,798, Mouse anti-hLamp2: DSHB H4B4-C, Mouse anti-Myc: DSHB 9E10, M2 Mouse anti-Flag antibody: Sigma F1804, Mouse anti-V5 antibody: Invitrogen 46 to 1157, Protein G-resin: Genescript L00209, GFP-Trap beads: ChromoTek, EZview Red Anti-HA Affinity Gel: Sigma E6779, EZview Red Anti-Flag Affinity Gel: Sigma F2426, Bafilomycin A1: Enzo BML-CM110 to 0100, Cycloheximide: Sigma 239,763, DDD86481: Aobious AOB13563, Alk12 was synthesized as previously described (63), Biotin Azide: APE x BIO/Fisher A8013. IRDye 680RD/800CW secondary antibodies were ordered from Invitrogen and LI-COR Biosciences. Alexa Fluor–conjugated secondary antibodies (488/594/647 nm) were purchased from Invitrogen.

### DNA constructs

Wild-type (WT) TMEM106B in pCMV-Sport6 and Flag-TMEM106B in p3xFLAG-CMV-7.1 were obtained and cloned as previously described (35). To generate the G2A TMEM106B mutant, we used the following forward (5′-CCCCGCGTGCCGACATGGCAAAGTCTCTTTCTCATTT-3′) and reverse (5′-AAATGAGAAAGAGACTTTGCCATGTCGGCACGCGGGG-3′) primers and performed site-directed mutagenesis. We then performed a second mutagenesis on the G2A TMEM106B plasmid to make the G2AK3R mutant using the following forward (5′-CCGCGTGCCGACATGGCAAGGTCTCTTTCTCATTTGCCTTTG-3′) and reverse (5′-CAAAGGCAAATGAGAAAGAGACCTTGCCATGTCGGCACGCGG-3′) primers. To overexpress WT TMEM106B and G2AK3R mutant with GFP tags, WT and G2AK3R TMEM106B were then PCR amplified using the following primers: forward for WT (5′-CTGGGATCCGCCACCATGGGAAAGTCTCTTTCTC-3′), forward for G2AK3R (5′-CTGGGATCCGCCACCATGGCAAGGTCT-3′), reverse for both (5′-GATAAGCTTTTACTGTTGTGGCTGAAG-3′). These PCR products and the pEGFP-N2 vector were then digested with BamHI and HindIII and ligated together. For overexpression of NMT1/2, we used the pCMV4a HA-NMT1 and pCMV4a HA-NMT2 plasmids which were cloned as previously described (46). Human cathepsin D was cloned as previously described (38). pCMV6-entry myc-hATP6AP1 was purchased from Origene. The 118 to 274 TMEM106B construct was a generous gift from Dr Peter Cherepanov (62). TMEM106B CRISPR lentivirus targeting exon 1 was cloned by ligating oligos (5′-CACCGGAGTCACATCTGAAAACATG-3′) and (5′-AAACCATGTTTTCAGATGTGACTCC-3′) into pLenti-CRISPR v2. pLenti-CRISPR v2 was purchased from Addgene.

### Click chemistry

Cells were incubated with 50 μM Alk12 for 6 h and then collected using ice-cold PBS. The cells were lysed using IP Lysis buffer (50 mM Tris pH 8.0, 150 mM NaCl, 1% Triton X-100, 0.1% deoxycholate, and protease inhibitor cocktail). Cell lysates were incubated for 20 min at 4 °C, sonicated, and centrifuged to remove insoluble proteins. Lysates were then incubated with homemade rabbit anti-TMEM106B antibodies (35) for 2 h, followed by protein G beads for an additional 2 h at 4 °C. After washing with the IP lysis buffer, the beads were incubated with 200 μM biotin-azide in 600 μM TBTA, 2 mM CuSO4, and 2 mM TCEP solution for half an hour. The reaction was quenched by adding SDS loading buffer and boiling for 2 min at 95 °C. To visualize myristoylated protein labeled with biotin, the membrane was incubated with IR 800-labeled streptavidin for 1 h, along with other secondary antibodies during Western blotting.

### Cell lysis, IP, Pull-downs, and Western blotting

Cells were either lysed with RIPA buffer (50 mM Tris pH 8, 150 mM NaCl, 1% Triton X-100, 0.1% deoxycholate, 1% SDS, protease inhibitors) or with IP lysis buffer and lysed as described above. For immunoprecipitations, lysates were incubated with anti-myc or GFP beads for 3 h on a nutator at 4 °C. The beads were then washed three times, and proteins were eluted by boiling the beads in SDS loading buffer with β-mercaptoethanol for 2 min at 95 °C. For inputs and cell lysates with TMEM106B overexpression, an equal volume of cell lysate and SDS loading buffer with β-mercaptoethanol was mixed before boiling at 95 °C for 2 min. For samples with endogenous TMEM106B, samples were mixed with SDS buffer without β-mercaptoethanol and were kept on ice. TMEM106B protein runs as a monomer when boiled and as a dimer when kept cold, as shown previously (27, 35). Proteins were separated using 12% SDS gels (15% for NTF and ICD detection). Gels were run at 80V for 10 min and then 120V for 1 h and 15 min. Gels were then transferred onto a PVDF membrane for 1 h at 300 mA. Following transfer, membranes were blocked with Odyssey blocking buffer for experiments using the anti-TMEM106B CTF antibody (clone Ab78), or with 5% milk in PBS for other antibodies, for 1 h. Membranes were incubated overnight with primary antibody at 4 °C, washed 3 times in TBST, and then incubated with secondary antibody at room temperature for at least 1 h. Membranes were washed three times in TBST again before scanning with the Odyssey scanner. The following primary antibodies were used for western blotting: Homemade Rabbit anti-TMEM106B ICD (a.a. 1–96) (35) 1:500; Human/Mouse chimeric anti-TMEM106B CTF (Clone Ab78) 1:1000; Proteintech Mouse anti-GAPDH (1:10,000); Sigma Mouse anti-HA (1:10,000); Homemade Rabbit anti-GFP (1:10,000); Sigma M2 mouse anti-FLAG (1:10,000); Invitrogen Mouse anti-V5 (1:1000).

### NMT reaction on TMEM106B N-terminal-derived synthetic peptides

50 mM Tris (pH 8.0), 50 μM NMT1 or PBS, 200 μM myristoyl-CoA, and 100 μM peptide in 50 μl reaction were incubated at 30  °C for 1 h. 50 μl of ACN was added to quench the reaction at RT for 30 min. The samples were then centrifuged at 17,000*g* for 10 min. The supernatants were transferred to a new glass tube, and then analyzed by LC-MS with a binary gradient of 0.1% acetic acid in water and 0.1% acetic acid in ACN over 12 min.

### Immunostaining and image analysis

In brief, coverslips were washed three times with cold PBS before fixation with 4% PFA for 15 min. Post-fixation cells were blocked and permeabilized with 0.05% saponin diluted in Odyssey blocking buffer for 30 min. The coverslips were then incubated with primary antibody diluted in the above buffer overnight at 4 °C. The coverslips were then washed three times with PBS before incubation with secondary antibodies diluted in the same buffer. Following three final PBS washes, the coverslips were then mounted onto slides with fluoromount. Cells were imaged using the 3i confocal microscope. Lysosome enlargement and co-localization were calculated using ImageJ FIJI. For lysosome enlargement, the diameter of the five largest lysosomes in each cell in an image was measured for at least 25 cells per coverslip. The experiment was repeated four times for statistical analysis. The following antibodies were used for immunostaining: Purified rabbit anti-TMEM106B ICD (1:300), mouse-anti hLAMP2 (1:300), Rat anti-Mouse LAMP1 (1:300), Hoescht (1:1000). For live cell staining, cells were transfected with TMEM106B as described previously. After 48 h, cells were washed 3 times with cold PBS and then incubated with human/mouse chimeric anti-TMEM106B CTF antibodies (1:300) diluted in Oddessey blocking buffer for 1 h at 4 °C. Then, the cells were washed again with cold PBS and fixed, permeabilized, and stained for intracellular TMEM106B using rabbit anti-TMEM106B ICD antibodies following the standard Immunostaining procedure.

### Statistical analysis

Statistical analysis was performed using GraphPad Prism 10. Data was analyzed using two different statistical tests. For comparisons where all the control samples were normalized to the value of 1, one sample *t*-tests were performed. For comparisons where everything was normalized to the average of the control samples, an unpaired student *t* test was performed for comparison between the two groups. For experiments with more than two groups, either one-way ANOVA or two-way ANOVA with Tukey *post hoc* tests was performed. For cell-surface TMEM106B quantifications with two groups, a ratio paired *t* test was used, for more than two groups a matched two-way ANOVA with *post hoc* comparisons was used. For all tests, we considered *p*-values<0.05 statistically significant ∗*p* < 0.05, ∗∗*p* < 0.01, ∗∗∗*p* < 0.001, ∗∗∗∗*p* < 0.0001. All *p*-values for one sample and unpaired t-tests reported are two-tailed *p*-values.

## Data availability

All data needed to evaluate the conclusions in the paper are present in the paper and/or the Supplementary Materials.

## Supporting information

This article contains supporting information.

## Ethics approval and consent to participate

All experimental procedures were approved by Cornell University.

## Consent for publication

All authors have given consent for publication.

## Conflict of Interest

The authors declare the following financial interests/personal relationships which may be considered as potential competing interests: H. L. is a founder and consultant for Sedec Therapeutics. AY, BG, IP, MAYC, and NFH are current or former employees of Alector, LLC and may have an equity interest in Alector, Inc. Several authors have patents related to TMEM106B-specific antibodies.
