# Supplementary material for: Myristoylation of TMEM106B by NMT1/2 regulates TMEM106B trafficking and turnover
Source: J Biol Chem. 2025 May 30;301(7):110322. doi: 10.1016/j.jbc.2025.110322 (PMC12268641; doi:10.1016/j.jbc.2025.110322)
Supplement: Supplemental Materials [file mmc1.pdf]

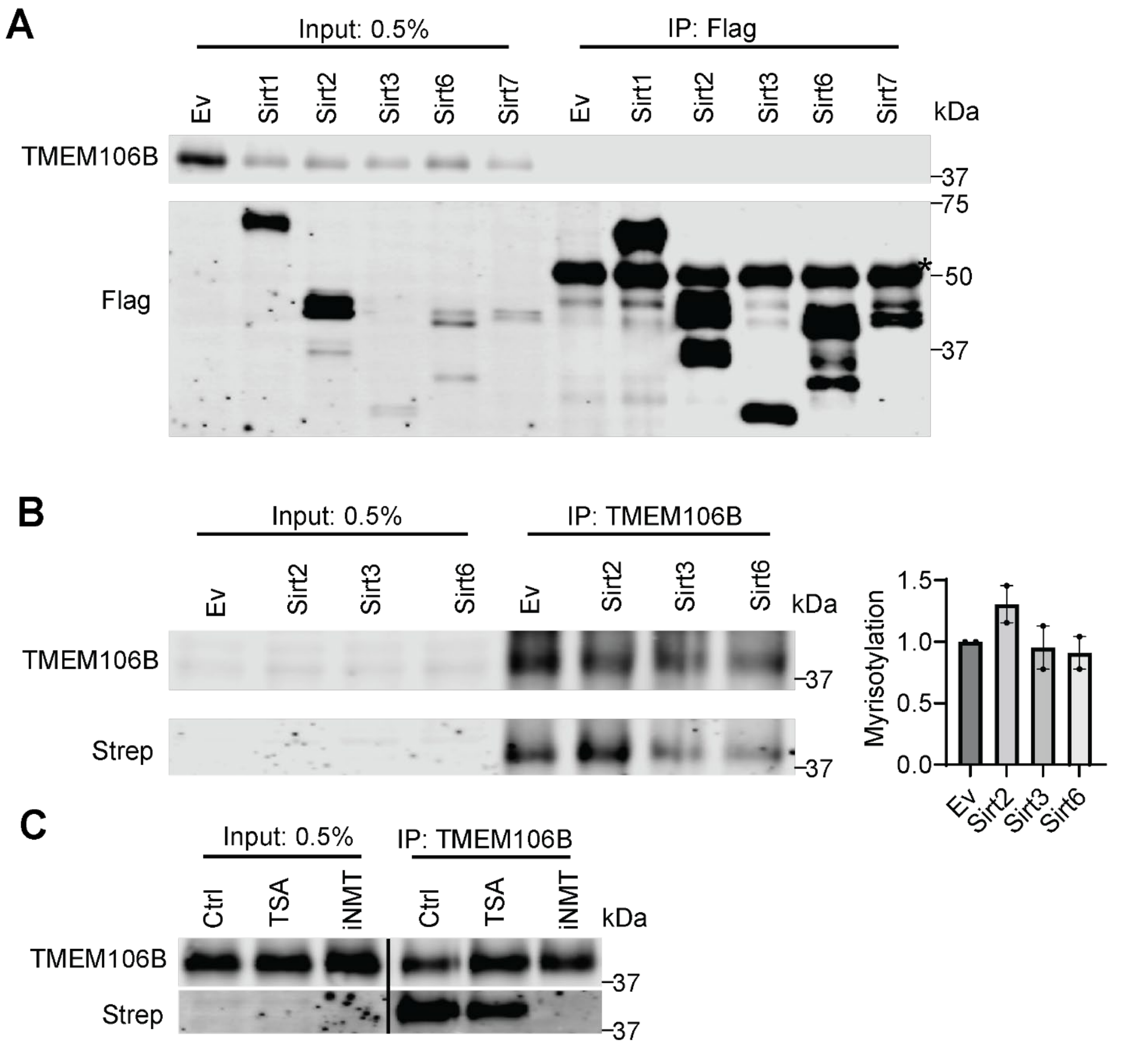

Supplemental Figure 1. **TMEM106B myristoylation is not modulated by Sirtuin/HDAC enzymes.** A) HEK239T cells were transfected un-tagged TMEM106B along with empty vector or flag-tagged Sirtuin 1, 2, 3,6, and 7. 48 hours after transfection the cells were lysed and immunoprecipitated with anti-Flag beads. Samples were analyzed via western blot. B) HEK293T cells were transfected with empty vector or flag-tagged Sirtuin 2, 3 or 6. 42 hours after transfection, HEK293T cells were incubated with myristic acid analog for an additional 6 hours. Endogenous TMEM106B was then immunoprecipitated and biotin click chemistry was performed. Proteins were separated using SDS-PAGE and blotted with anti-TMEM106B antibodies or fluorescently labeled streptavidin. The intensity of biotinylated TMEM106B band was quantified and normalized to total TMEM106B levels probed by anti-TMEM106B antibodies (n=2). C) HEK293T cells were transfected with WT TMEM106B. 24 hours after transfection, the cells were treated with either vehicle control NMT inhibitor, or TSA (an HDAC inhibitor). 48 hours after transfection TMEM106B was immunoprecipitated and myristoylation assays were performed as in b). *indicates non-specific band likely corresponding to heavy IgG eluted from the anti-flag beads. Data represent mean +/- SEM; ns, not-significant; *, $p<0.05$; **, $p<0.01$; ***, $p<.001$; ****, $p<.0001$

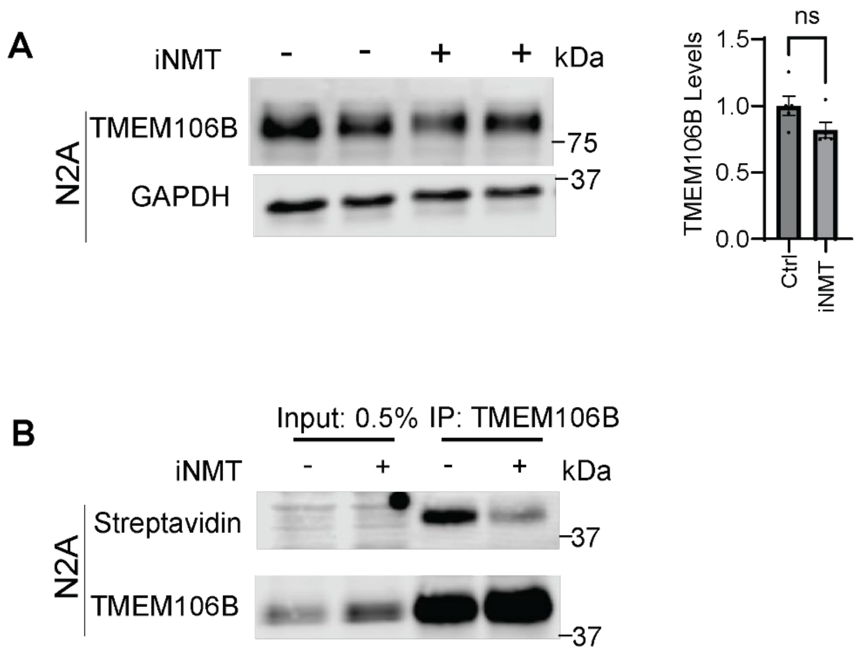

***Supplemental Figure 2.* NMT inhibitor does not reduce T106B levels in N2A cells.** A) N2A cells were either treated with vehicle control or with iNMT for 24 hours before being lysed and analyzed via western blot (n=4, unpaired t-test). B) N2A, cells were incubated with myristic acid analog with and without iNMT treatment. TMEM106B was then immunoprecipitated and biotin click chemistry was performed. Proteins were separated using SDS-PAGE and blotted with anti-TMEM106B antibodies or fluorescently labeled streptavidin. Data represent mean +/- SEM; ns, not-significant; *, p<0.05; **, p<0.01; ***, p<.001; ****, p<.0001

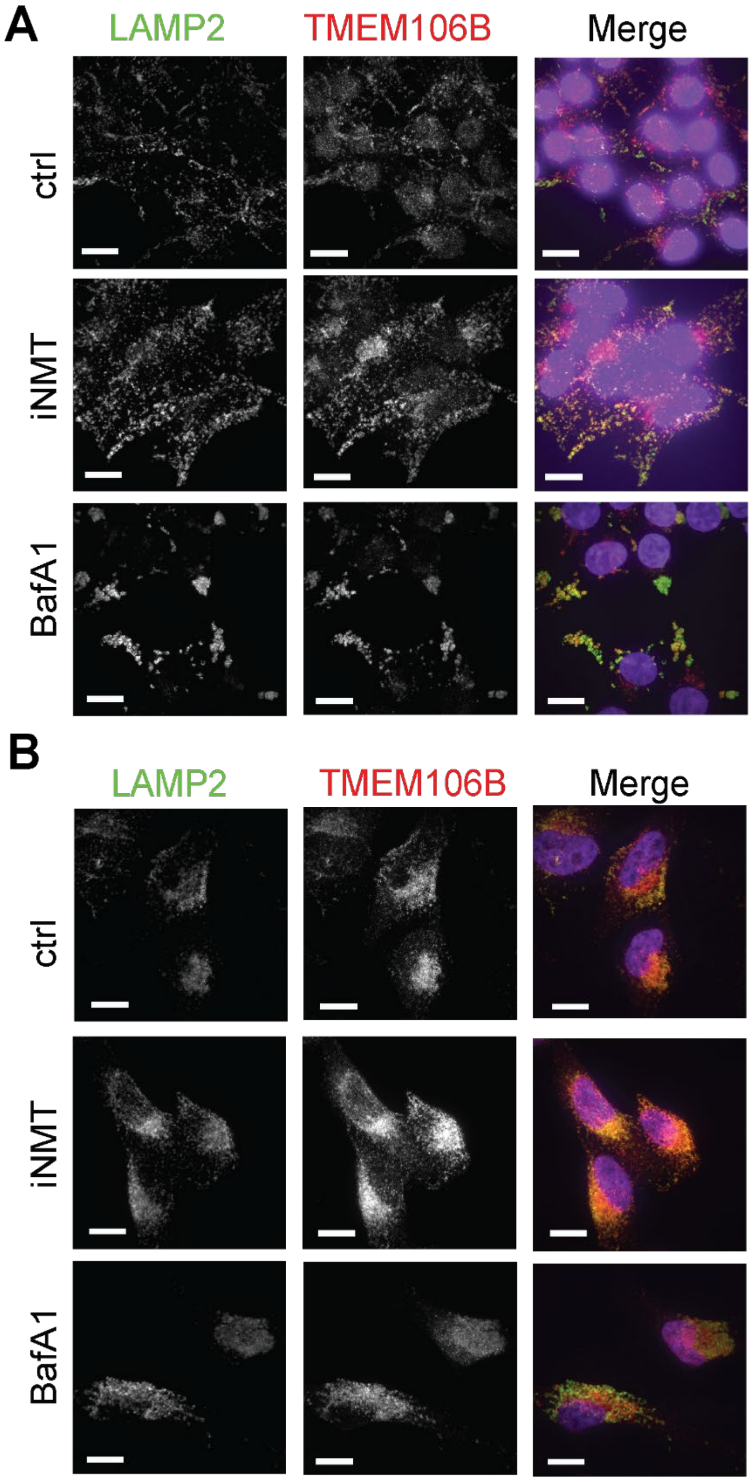

Supplemental Figure 3. **Inhibition of NMT does not significantly impair TMEM106B lysosome trafficking.** A) HEK293T cells treated with NMT inhibitor (iNMT) for 24 hours or BafA1 for 16 hours. Cells were fixed and stained with anti-LAMP2 and TMEM106B ICD antibodies. B) Hela cells treated with iNMT or BafA1 for 24 hours before immunostaining with anti-LAMP2 and TMEM106B ICD antibodies. Scale bar=10uM

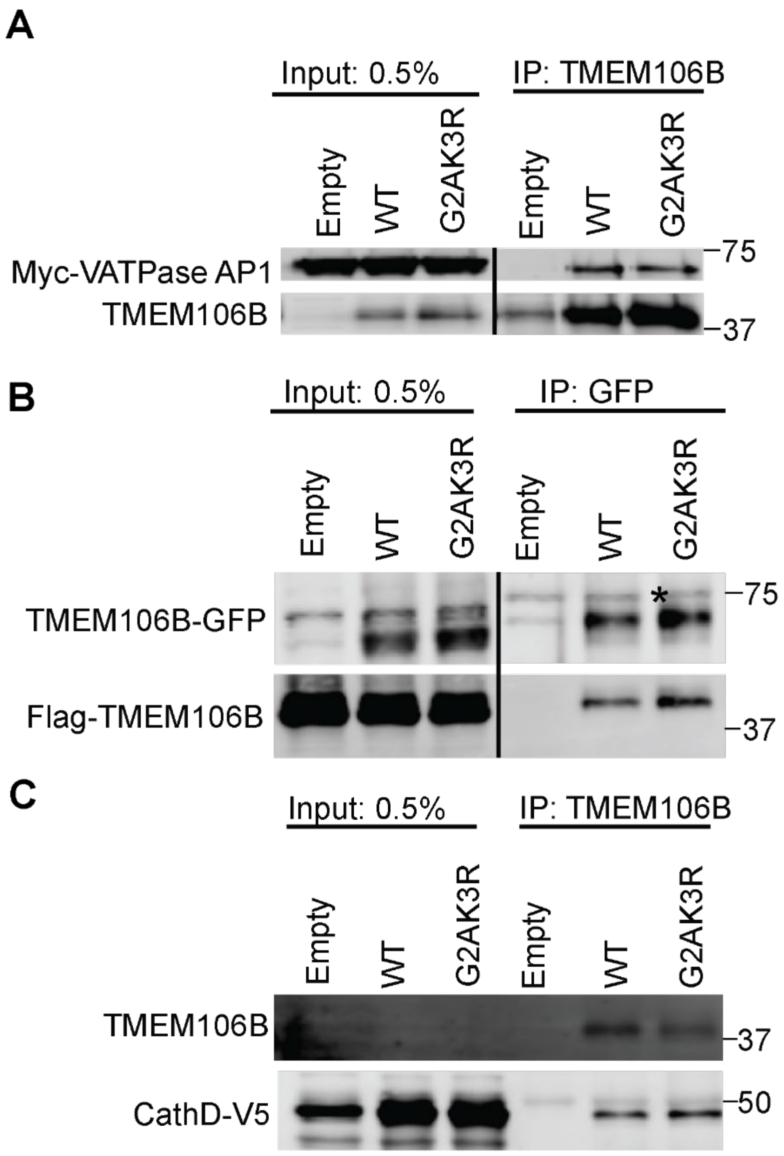

***Supplemental Figure 4*. TMEM106B myristoylation does not affect its binding to previously identified binding partners**. A) HEK239T cells were transfected with Myc-VATPaseAP1 along with empty vector, WT TMEM106B, or G2AK3R TMEM106B. 48 hours after transfection the cells were lysed and immunoprecipitated with anti-TMEM106B antibodies. Samples were analyzed via western blot. B) HEK239T cells were transfected with Flag-TMEM106B along with empty vector, WT TMEM106B-GFP, or G2AK3R TMEM106B-GFP. 48 hours after transfection the cells were lysed and immunoprecipitation was performed with GFP beads. Samples were analyzed via western blot. C) HEK239T cells were transfected with Cathepsin D-V5 along with empty vector, WT TMEM106B, or G2AK3R TMEM106B. 48 hours after transfection the cells lysed and immunoprecipitated with anti-TMEM106B antibodies. Samples were analyzed via western blot. *Indicates non-specific band in the GFP blot.

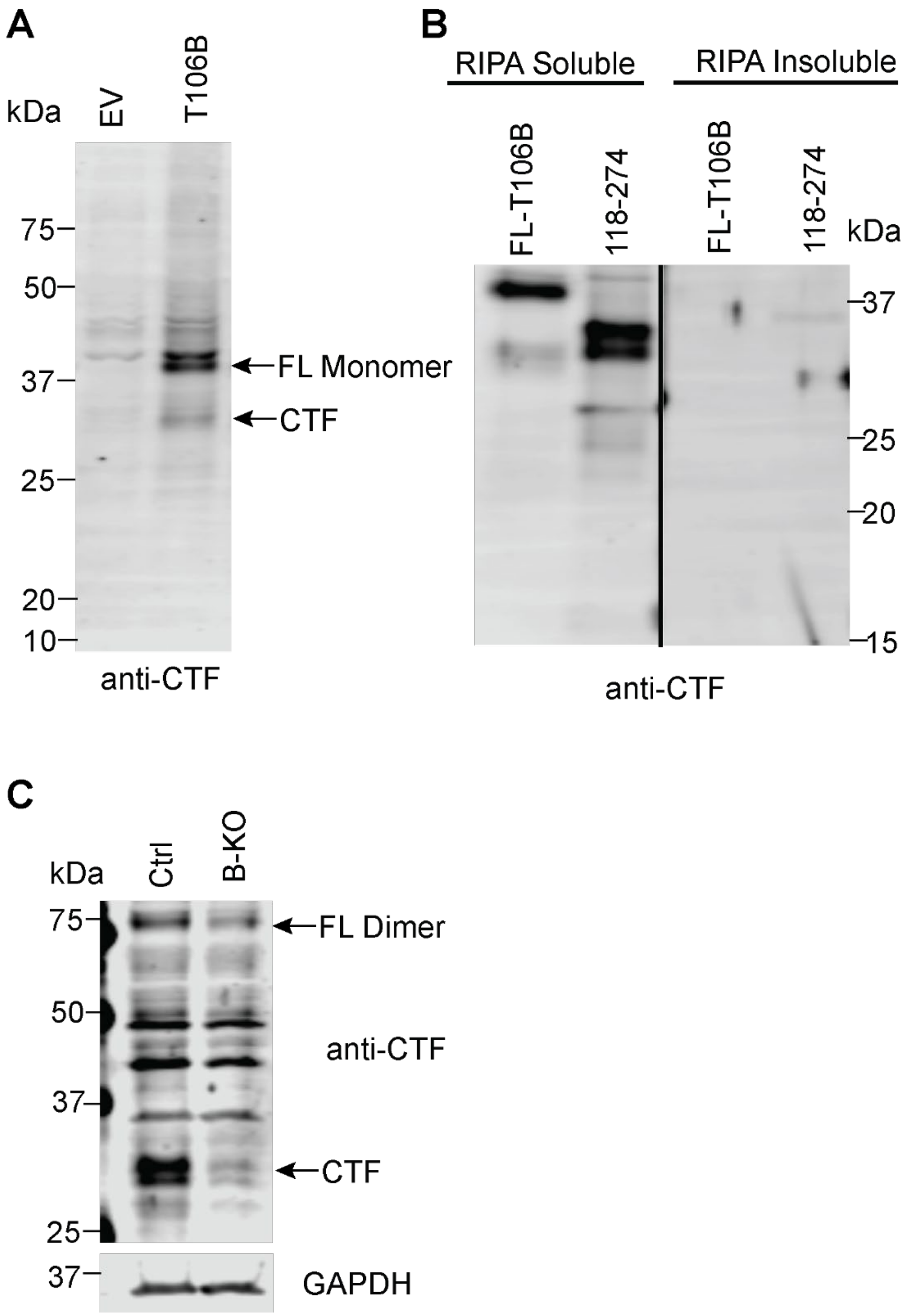

Supplemental Figure 5. **Validation of anti-TMEM106B CTF antibody (Alector Clone Ab78) for western blotting applications.** A) HEK293T cells transfected with empty vector or TMEM106B for 48 hours before being lysed. Western blot analysis was performed with anti-TMEM106B CTF antibody. B) HEK293T cells were transfected with full-length TMEM106B, or IgK-118-274 TMEM106B for 48 hours and then lysed with RIPA buffer. The RIPA soluble and insoluble fractions were analyzed via western blotting. C) Pooled CRISPR Ctrl and TMEM106B KO HEK293T cells were lysed and compared via western blot to validate Ab78.

Supplementary Fig. 6 **Uncropped Western Blots**

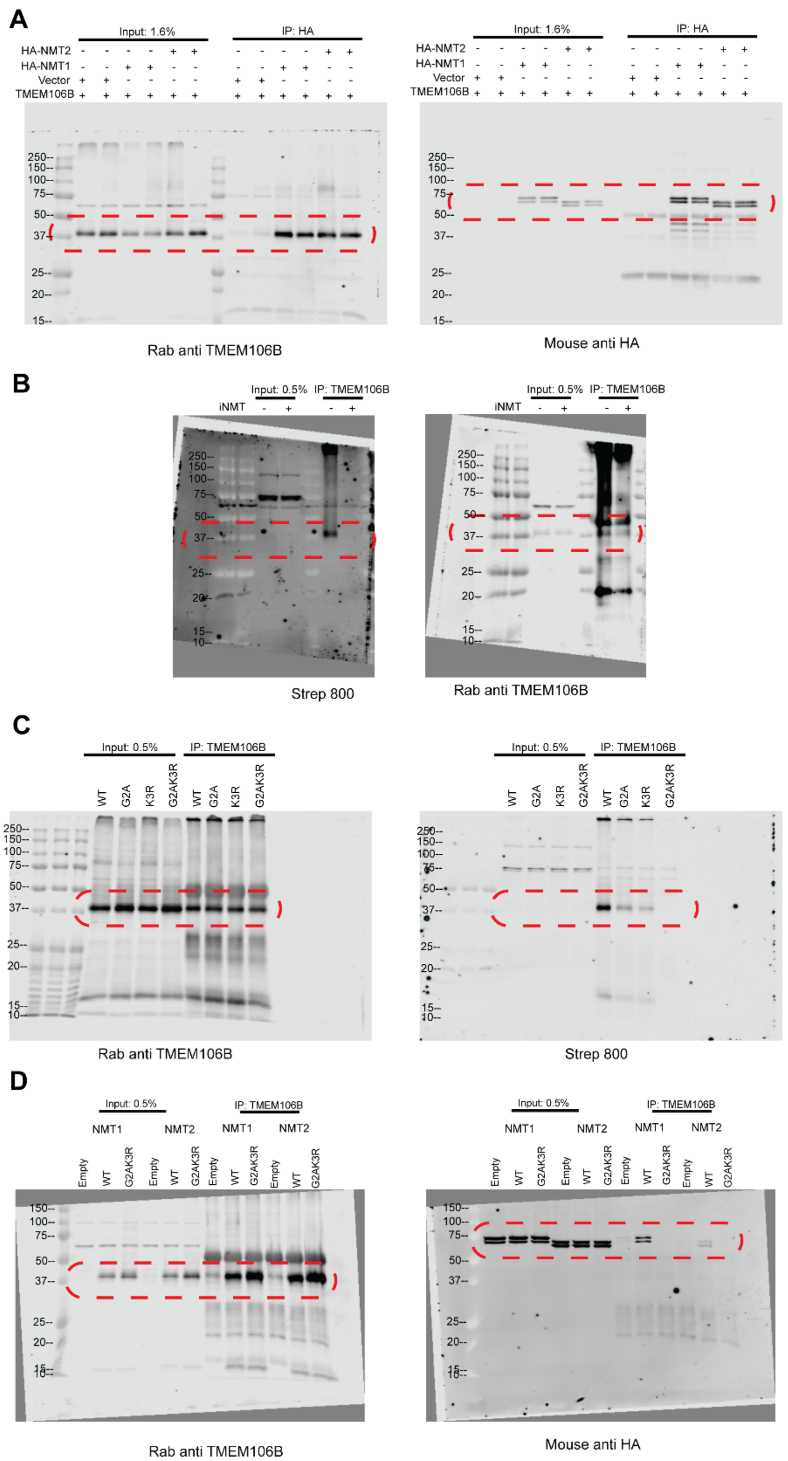

Figure 1

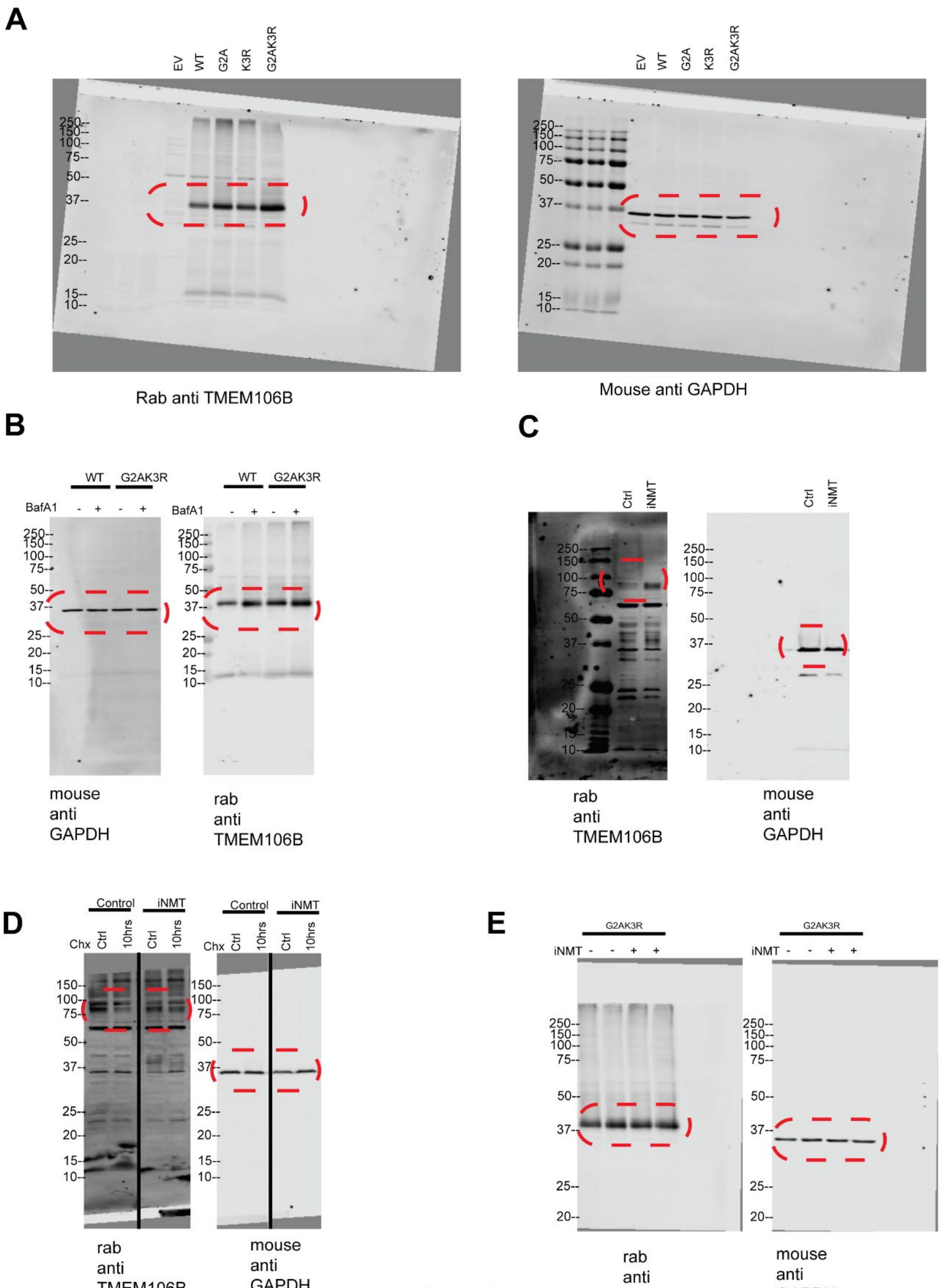
A
EV
WT
G2A
K3R
G2AK3R
250--
150--
100--
75--
50--
37--
25--
20--
15--
10--
Rab anti TMEM106B
Mouse anti GAPDH
B
WT
G2AK3R
BafA1
- + - +
mouse anti GAPDH
rab anti TMEM106B
C
Ctrl
iNMT
rab anti TMEM106B
mouse anti GAPDH
D
Control
iNMT
Chx
Ctrl
10hrs
rab anti TMEM106B
mouse anti GAPDH
E
G2AK3R
iNMT
- - + +
rab anti TMEM106B
mouse anti GAPDH

Figure 3

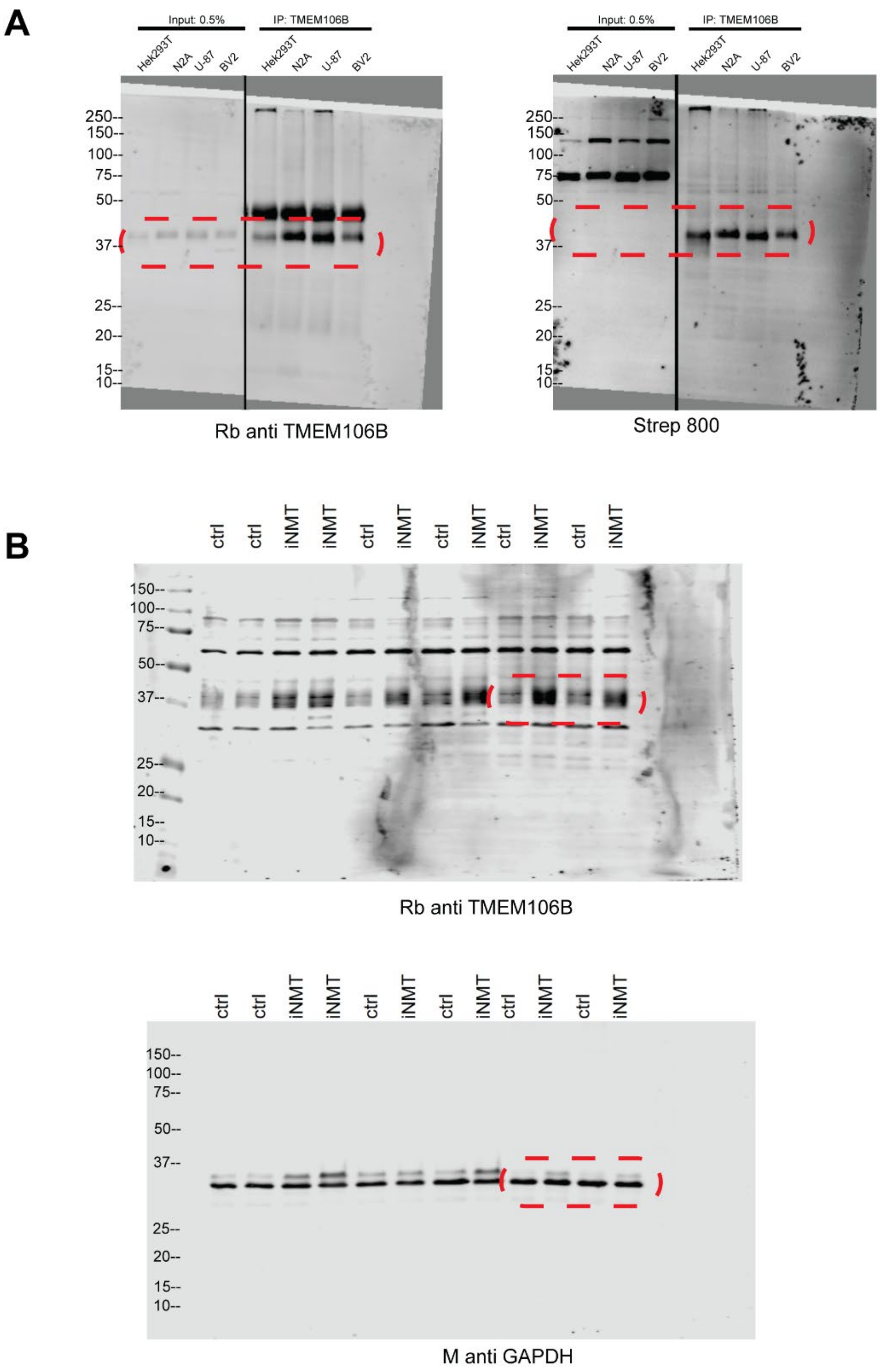
A
Input: 0.5%
IP: TMEM106B
Hek293T
N2A
U-87
BV2
250--
150--
100--
75--
50--
37--
25--
20--
15--
10--
Rb anti TMEM106B
Strep 800
B
ctrl
iNMT
Rb anti TMEM106B
M anti GAPDH

Figure 4

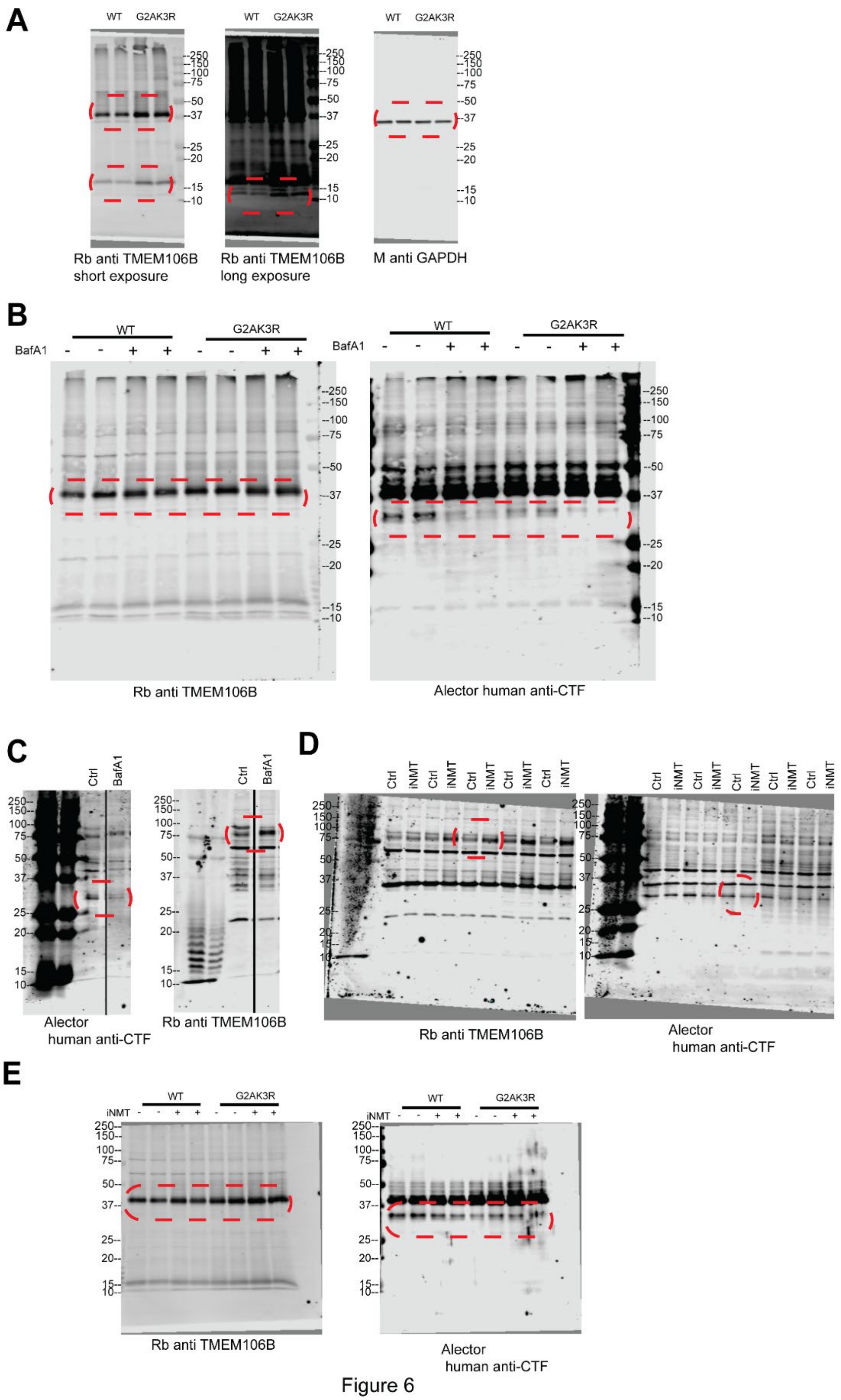

A
WT
G2AK3R
Rb anti TMEM106B short exposure
Rb anti TMEM106B long exposure
M anti GAPDH
B
BafA1
Rb anti TMEM106B
Alector human anti-CTF
C
Ctrl
BafA1
Alector human anti-CTF
Rb anti TMEM106B
D
Ctrl
iNMT
Rb anti TMEM106B
Alector human anti-CTF
E
iNMT
Rb anti TMEM106B
Alector human anti-CTF

Figure 6

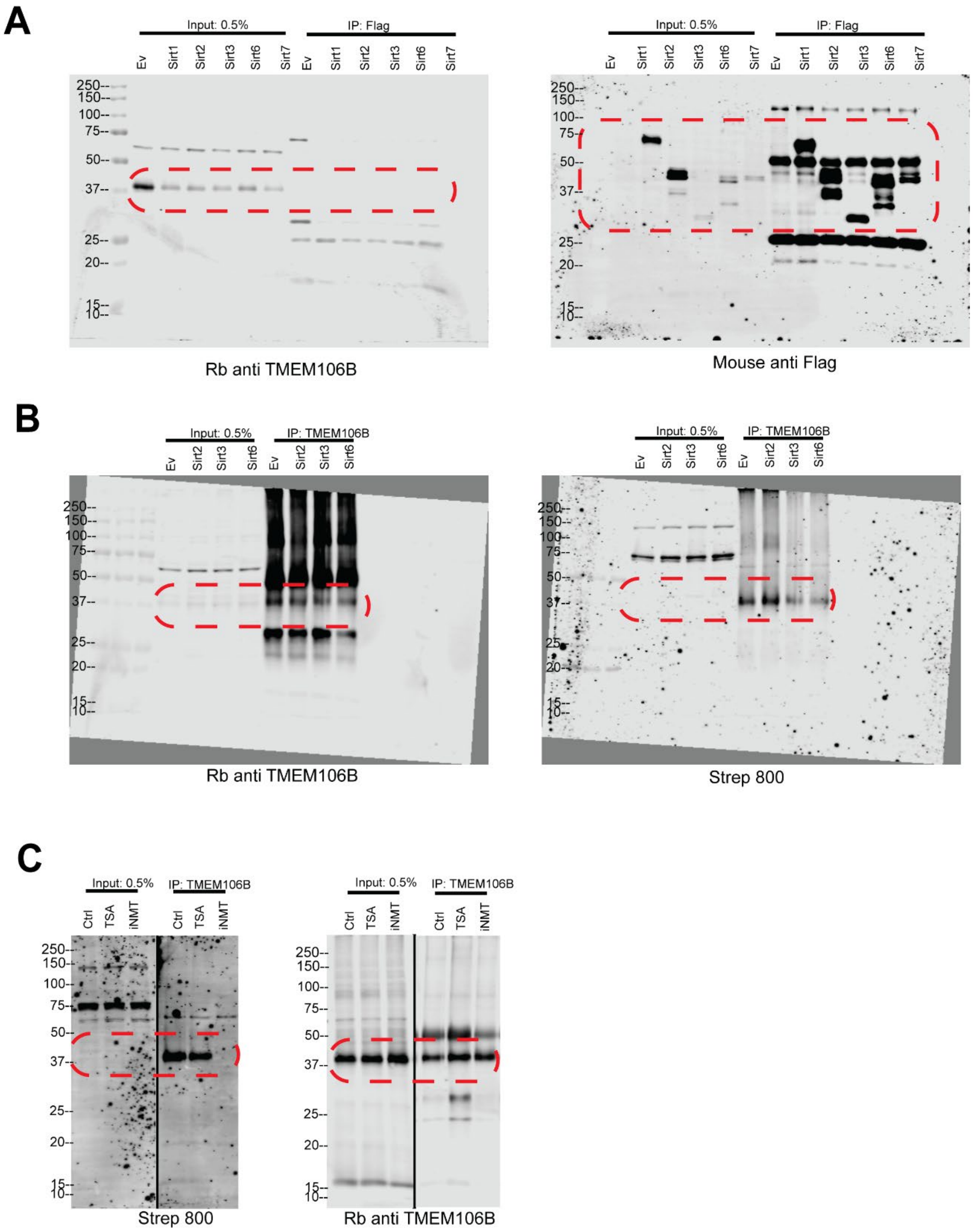
A
Input: 0.5%
IP: Flag
Ev Sirt1 Sirt2 Sirt3 Sirt6 Sirt7 Ev Sirt1 Sirt2 Sirt3 Sirt6 Sirt7
250-- 150-- 100-- 75-- 50-- 37-- 25-- 20-- 15-- 10--
Rb anti TMEM106B
Input: 0.5%
IP: Flag
Ev Sirt1 Sirt2 Sirt3 Sirt6 Sirt7 Ev Sirt1 Sirt2 Sirt3 Sirt6 Sirt7
250-- 150-- 100-- 75-- 50-- 37-- 25-- 20-- 15-- 10--
Mouse anti Flag
B
Input: 0.5%
IP: TMEM106B
Ev Sirt2 Sirt3 Sirt6 Ev Sirt2 Sirt3 Sirt6
250-- 150-- 100-- 75-- 50-- 37-- 25-- 20-- 15-- 10--
Rb anti TMEM106B
Input: 0.5%
IP: TMEM106B
Ev Sirt2 Sirt3 Sirt6 Ev Sirt2 Sirt3 Sirt6
250-- 150-- 100-- 75-- 50-- 37-- 25-- 20-- 15-- 10--
Strep 800
C
Input: 0.5%
IP: TMEM106B
Ctrl TSA iNMT Ctrl TSA iNMT
250-- 150-- 100-- 75-- 50-- 37-- 25-- 20-- 15-- 10--
Strep 800
Input: 0.5%
IP: TMEM106B
Ctrl TSA iNMT Ctrl TSA iNMT
250-- 150-- 100-- 75-- 50-- 37-- 25-- 20-- 15-- 10--
Rb anti TMEM106B

Figure S1

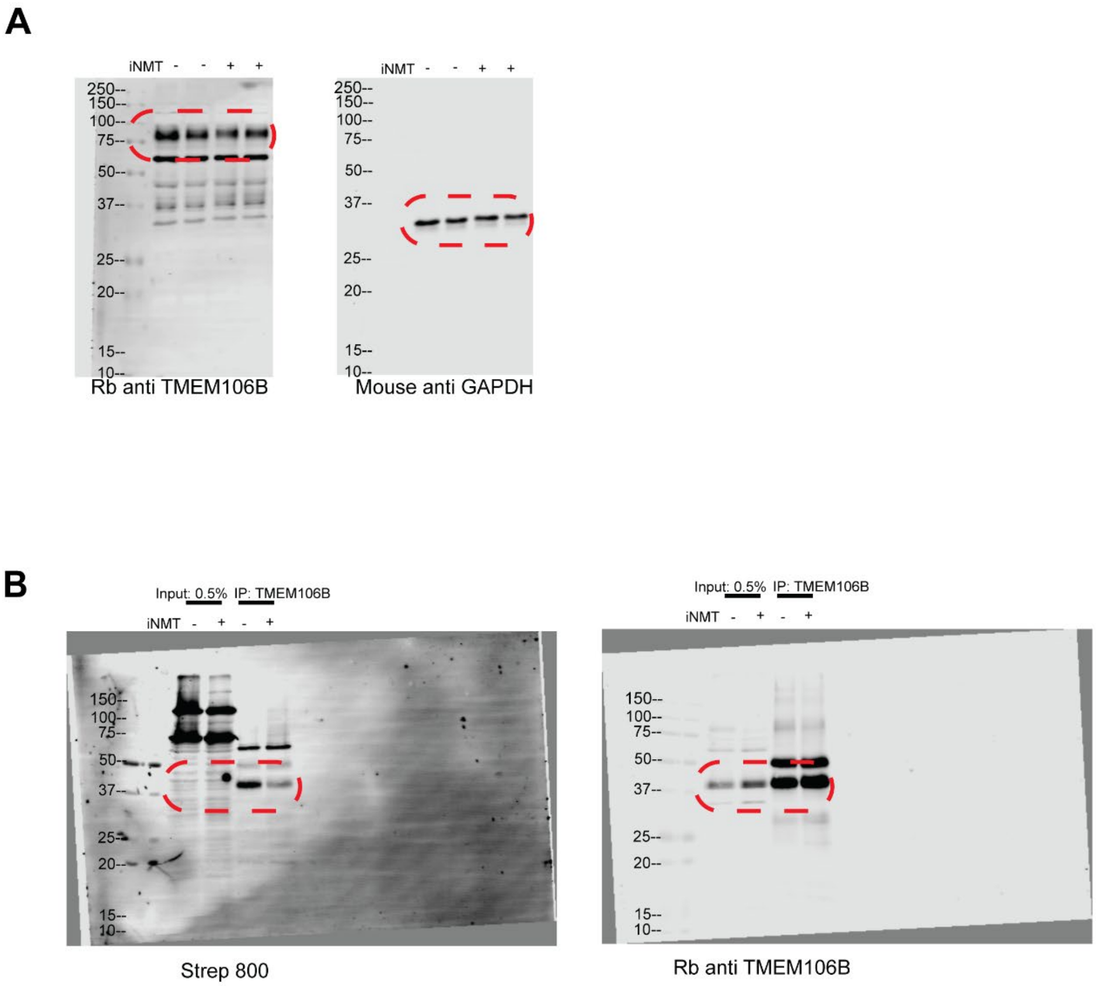
A
iNMT - - + +
250--
150--
100--
75--
50--
37--
25--
20--
15--
10--
Rb anti TMEM106B
iNMT - - + +
250--
150--
100--
75--
50--
37--
25--
20--
15--
10--
Mouse anti GAPDH
B
Input: 0.5% IP: TMEM106B
iNMT - + - +
150--
100--
75--
50--
37--
25--
20--
15--
10--
Strep 800
Input: 0.5% IP: TMEM106B
iNMT - + - +
150--
100--
75--
50--
37--
25--
20--
15--
10--
Rb anti TMEM106B

Figure S2

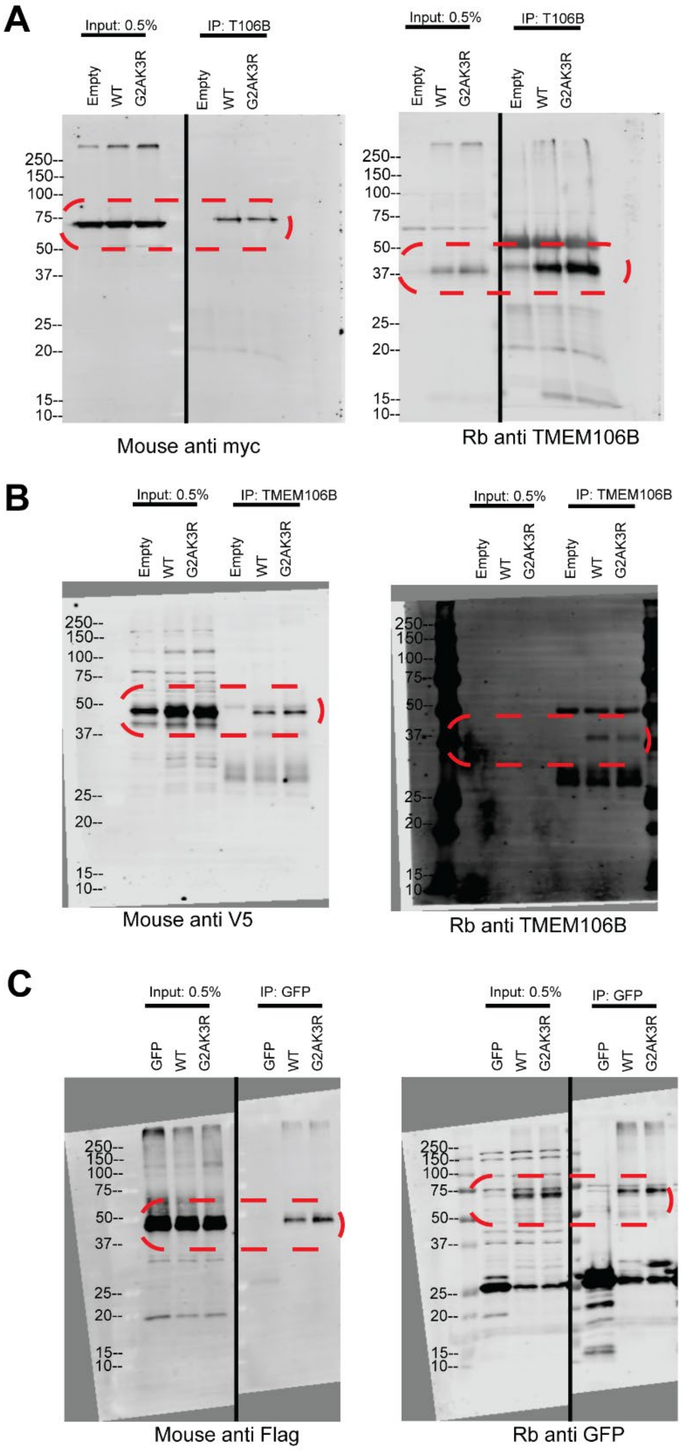

A
Input: 0.5%
IP: T106B
Empty
WT
G2AK3R
250--
150--
100--
75--
50--
37--
25--
20--
15--
10--
Mouse anti myc
Rb anti TMEM106B
B
Input: 0.5%
IP: TMEM106B
Mouse anti V5
Rb anti TMEM106B
C
Input: 0.5%
IP: GFP
GFP
WT
G2AK3R
Mouse anti Flag
Rb anti GFP

Figure S4

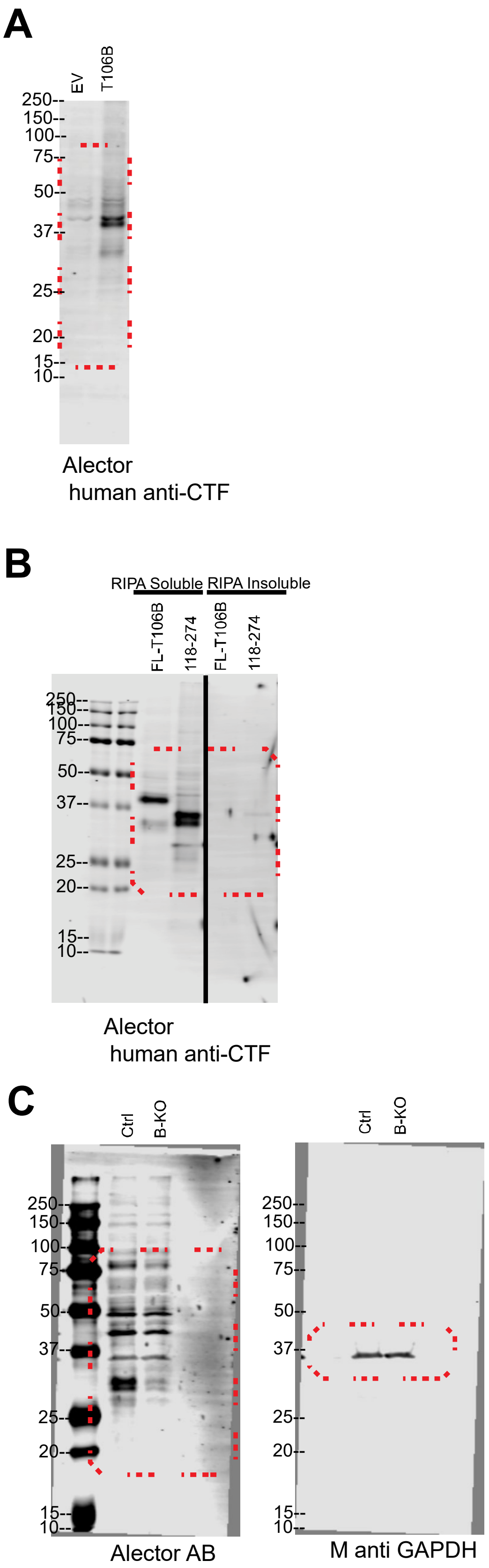
A
EV
T106B
250--
150--
100--
75--
50--
37--
25--
20--
15--
10--
Alector
human anti-CTF
B
RIPA Soluble
RIPA Insoluble
FL-T106B
118-274
FL-T106B
118-274
250--
150--
100--
75--
50--
37--
25--
20--
15--
10--
Alector
human anti-CTF
C
Ctrl
B-KO
250--
150--
100--
75--
50--
37--
25--
20--
15--
10--
Alector AB
Ctrl
B-KO
250--
150--
100--
75--
50--
37--
25--
20--
15--
10--
M anti GAPDH

Figure S5
